# Development and evaluation of novel zein-based artemisinin sustained-release formulation for treating drug-resistant malaria

**DOI:** 10.1128/mbio.03696-25

**Published:** 2026-01-12

**Authors:** Yijie Wang, Xinyu Yu, Xinyu Zhang, Xiaohui He, Yongxin Tang, Ling Fang, Richard Culleton, Qingfeng Zhang, Weifu Dong, Jun Cao

**Affiliations:** 1 The Key Laboratory of Synthetic and Biological Colloids, Ministry of Education, School of Chemical and Material Engineering, Jiangnan University 631874, Wuxi, China; 2 Center for Global Health, School of Public Health, Nanjing Medical University 572407, Nanjing, China; 3 National Health Commission Key Laboratory of Parasitic Disease Control and Prevention, Jiangsu Provincial Key Laboratory on Parasite and Vector Control Technology, Jiangsu Institute of Parasitic Diseases 608817, Wuxi, China; 4 Xishan People’s Hospital of Wuxi City, Wuxi Branch of Zhongda Hospital Southeast University 699214 , Wuxi, China; 5 Division of Parasitology, Proteo-Science Centre, Ehime University 12760, Matsuyama, Ehime, Japan; 6 Laboratory of Molecular Parasitology, State Key Laboratory of Cardiology and Research Center for Translational Medicine, Shanghai East Hospital; Clinical Center for Brain and Spinal Cord Research, School of Medicine, Tongji University 481875, Shanghai, China; 7 Public Health Research Center, Jiangnan University 66374, Wuxi, China; Iowa State University, Ames, Iowa, USA

**Keywords:** *Plasmodium*, artemisinin resistance, zein-based nanoparticle, sustained release

## Abstract

Artemisinin antimalarial drugs initially exhibited remarkable efficacy against *Plasmodium falciparum*. However, their poor solubility and low bioavailability necessitate high doses and lead to an extremely short *in vivo* half-life. These limitations not only drive the emergence of drug-resistant *Plasmodium* strains but also compromise long-term therapeutic outcomes. Herein, we report a zein-based sustained release formulation, wherein zein, a natural maize protein, serves as a biocompatible nanocarrier to effectively encapsulate artemisinin (ART). Notably, this nanocarrier formulation achieves a 200-fold enhancement in ART’s water solubility, addressing a key bottleneck of ART-based therapies. *In vitro* assays confirm that the zein-based formulation allows for the sustained release of ART, which could help maintain therapeutic concentrations over extended periods and displayed different release rates and good dispersibility in both acid and basic environments. Importantly, *in vitro* evaluations also demonstrate that the nanoformulation exerts potent inhibitory effects against ART-resistant *P. falciparum* strains in both ring survival assay and recrudescence assay, attributed to the sustained maintenance of effective ART concentrations. *In vivo* studies, utilizing both rodent malaria models and humanized erythrocyte mouse models, further validate the nanoformulation’s therapeutic potential. The zein nanocarrier significantly prolongs ART’s *in vivo* half-life via its sustained-release capability, thereby maintaining effective blood concentrations over an extended duration. Compared to free ART, the nanoformulation exhibits superior efficacy in reducing parasitemia, preventing malaria recrudescence, and, most notably, overcoming ART resistance in drug-resistant *Plasmodium* infections. Collectively, these findings establish the zein-based nanocarrier as a promising strategy to optimize ART-based therapies by addressing solubility and pharmacokinetic limitations while effectively combating drug-resistant malaria.

**IMPORTANCE:**

Half of the world’s population is at risk of malaria infection, and artemisinin (ART) turns out to be a powerful medicine for malaria control. The rapid emergence and global spread of resistance to ART have led to a significantly increasing clinical treatment failure rate worldwide. A critical limitation of ART is its extremely short blood half-life (~1 h), which results in rapid declines in plasma drug concentrations below therapeutic thresholds. Some parasites may switch into a “dormant” form, which is less sensitive to ART, resulting in recrudescence following treatment. Thus, developing a sustained-release formulation provides a promising solution to prolong the *in vivo* half-life of ART. Additionally, its relatively low solubility restricts its *in vivo* bioavailability, primarily due to the limited dissolution and absorption of the compound in aqueous biological environments. In this study, we prepared a zein-based sustained-release formulation of ART for oral and intraperitoneal administration. Our results indicate that this zein-based sustained release nanoformulation not only significantly improves ART’s water solubility (a key barrier to its bioavailability) but also extends its *in vivo* half-life via controlled drug release. Importantly, the prolonged half-life ensures sustained therapeutic ART concentrations, directly enhancing the formulation’s ability against ART-resistant *P. falciparum* strains. Collectively, these results highlight the formulation’s substantial potential for clinical application in improving ART-based antimalarial therapy.

## INTRODUCTION

Half of the world’s population is at risk of malaria infection (1, 2). According to the WHO 2024 World Malaria Report, there were about 263 million cases and more than 597,000 deaths due to malaria worldwide in 2023 (3). As the protective efficacy and durability of currently approved vaccines still remain controversial, drug treatment remains the most effective and cost-efficient strategy for malaria control, particularly in high-burden regions such as Sub-Saharan Africa (4). However, *P. falciparum* has increasingly developed widespread resistance to most first-line antimalarial agents, which has emerged as a major bottleneck hampering global efforts toward malaria elimination (5–7). Thus, the global emergence and rapid spread of drug resistance have made it imperative to identify novel drug targets and discover new antimalarial agents.

Among them, artemisinin and its derivatives are currently recognized as the most effective first-line antimalarial drugs worldwide due to rapid parasite clearance efficiency and a favorable safety profile with minimal adverse effects (8–11). Thus, artemisinin-based therapies (ACTs) are recommended by the WHO as the preferred front-line treatment for malaria. It has been validated that ART specifically targets the asexual erythrocytic stage of malaria parasites, which is the key stage responsible for clinical manifestations of the disease (12, 13). However, ART exhibits an extremely short blood circulation half-life (~1–2 h), which frequently limits its ability to achieve complete parasite eradication (14, 15). Additionally, parasites may also enter a “dormant” or “quiescent” state under drug pressure, during which they become less sensitive to ART and its derivatives. This could directly result in recrudescence of the disease post-treatment (16–18). Furthermore, ART and its derivatives possess poor amphiphilic solubility (i.e., low solubility in both aqueous and lipid environments) and inherent chemical instability, which are factors affecting bioavailability and applicability (19). In detail, this is partly attributed to its intrinsic structural characteristics, specifically the dominant hydrophobic skeleton with insufficient hydrophilic groups and a dense crystal structure that impedes water molecule infiltration (20, 21). The core structure of ART consists of a sesquiterpene lactone ring, primarily composed of hydrophobic moieties, such as hydrocarbon chains and isopropyl groups. Its hydrophilic groups are not only scarce but also weak in hydrophilicity, making this the fundamental driver of its poor water solubility. Meanwhile, the dense arrangement of ART molecules in the crystalline state further exacerbates this poor solubility by preventing water molecules from penetrating into the crystal interior. To address this challenge, extensive efforts have been devoted to solubility improvement, including structural modification and cocrystal formation as strategies designed to disrupt the dense crystal structure of ART and enhance its interaction with water molecules. These physicochemical drawbacks significantly impair their bioavailability and clinical applicability by causing uneven distribution of the drug in the body, resulting in lower concentrations at target cells or tissues and diminishing its antimalarial efficacy (22–24). Consequently, higher doses may be necessary to maintain therapeutic drug concentrations at target sites, which in turn elevates the potential risk of adverse effects. Furthermore, the extensive and continuous clinical application of ART and its derivatives has led to the emergence and spread of drug-resistant malaria strains in various regions, challenging the therapeutic efficacy of these antimalarials in those areas (25, 26). The emergence of antimalarial resistance not only leads to elevated mortality rates and increased medical burdens for patients but also imposes substantial economic losses through reduced productivity (27). To solve the issue of drug resistance, several strategies have been proposed, including the rational use of ACTs, the development of improved drug formulations with enhanced pharmacokinetic profiles, and the discovery and clinical translation of novel antimalarial candidates (8, 11, 27).

The development of sustained-release formulations represents a promising therapeutic strategy to address the challenges associated with ART’s suboptimal bioavailability and the dormant state of malaria parasites (23, 28–30). Sustained-release formulations not only improve the solubility and dispersibility of ART (a key limitation of the free drug) but also enable its controlled and gradual release. When malaria parasites enter a dormant state and exhibit reduced sensitivity to ART, these sustained-release carriers help maintain stable, therapeutic drug concentrations in the circulation over an extended period. This ensures that the drug remains bioavailable to exert its antimalarial effect immediately upon parasite re-growth, thereby preventing post-treatment recrudescence. Additionally, a sustained-release approach allows for the maintenance of optimal drug concentrations at target sites over time, thus reducing the required dosage of ART for effective treatment and minimizing the risk of dose-dependent toxicity and side effects (30). Currently, various drug delivery carriers have been utilized as ART sustained-release formulations, including polymer carriers, nanocarriers, and liposomes, each with its own advantages and limitations (22, 31–34). For instance, while nanocarriers could improve drug bioavailability and stability, concerns regarding their long-term biocompatibility and biosafety persist; liposomes exhibit excellent biocompatibility and biomimetic properties that facilitate drug accumulation; however, they often suffer from poor colloidal stability and rapid clearance *in vivo*.

Among the diverse materials explored for antimalarial drug delivery, zein, a natural prolamin protein predominantly extracted from corn kernels, has emerged as a promising candidate (35–38). This plant-based protein exhibits excellent biocompatibility and biodegradability, with its degradation products (primarily amino acids) being non-toxic and readily metabolized by the human body. Additionally, zein is environmentally friendly and exhibits a significantly lower carbon footprint compared to animal-derived biomaterials. Notably, a key advantage of zein lies in its unique chemical structure and intrinsic amphiphilic physical properties, which enable it to self-assemble into stable colloidal structures in aqueous environments (39–41). These structural characteristics make zein an ideal carrier for hydrophobic drugs, including ART. It can efficiently encapsulate lipophilic compounds within its hydrophobic core, which not only markedly enhances their solubility but also protects them from chemical degradation. Thus, this encapsulation strategy can effectively improve the drug’s bioavailability, ensure a sustained release profile, and optimize the overall therapeutic efficacy.

In this study, we hypothesized that upon loading on the zein-based nanoparticles, either the *in vivo* pharmacokinetics profile or the solubility of ART could be significantly improved due to zein’s unique chemical structure and physical properties. These improvements are expected to enhance ART’s bioavailability and enable efficient elimination of drug-resistant parasites. Thus, we prepared a zein-based sustained-release formulation of ART for oral and intravenous administration, and both improved dissolution and reduced hemolysis of ART were achieved. The formulation was characterized in terms of size, polydispersity, and encapsulation efficiency. Subsequently, the physical and chemical stability, release characteristics, and biocompatibility were evaluated. *In vitro* antimalarial activity was tested against drug-sensitive and drug-resistant parasite strains using various phenotype assays, including growth inhibition, ring survival, and recrudescence assays, confirming robust efficacy against resistant strains. Additionally, the pharmacokinetic profile for the sustained release formulation was determined to investigate whether the proposed formulation can extend ART’s blood circulation half-life and improve bioavailability compared to free ART. To further validate the translational potential of the formulation, *in vivo* antimalarial efficacy was comprehensively assessed using both a standard rodent malaria model (*Plasmodium berghei* and *Plasmodium chabaudi*) and a humanized mouse model infected with *P. falciparum*. The results demonstrated that the zein-based formulation significantly improved the survival rate of infected mice compared to free ART treatment. More importantly, the sustained therapeutic drug concentrations achieved by the formulation effectively suppressed parasite regrowth, markedly reducing post-treatment recrudescence, even in models infected with ART-resistant strains. These *in vivo* findings directly validate that the enhanced solubility, prolonged half-life, and elevated bioavailability of the zein-based ART formulation translate to superior therapeutic outcomes, addressing the key challenge of ART resistance.

## RESULTS

**Fig 1:**
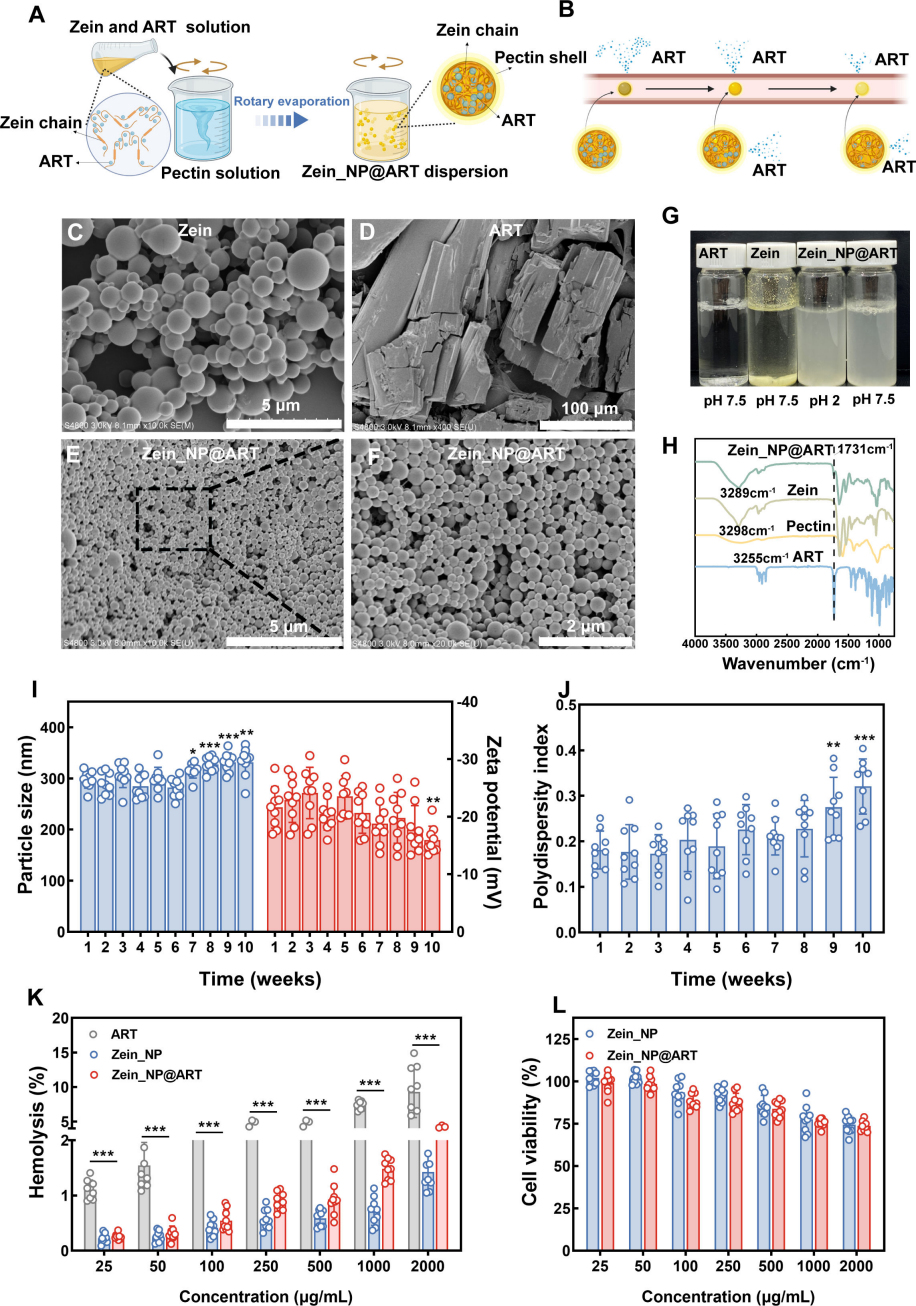
Synthesis and characterization of Zein_NP@ART. (**A**) Schematic illustration of the formation process of ART-loaded zein nanoparticle (Zein_NP@ART) dispersion. (**B**) Schematic representation of the sustained release behavior of ART from Zein_NP@ART in the physiological environment. SEM images of zein colloid (**C**), free ART (**D**), and Zein_NP@ART (**E-F**), depicting the morphological characteristics of each sample. (**G**) Solubility of Zein_NP and Zein_NP@ART dispersions under pH 2 and pH 7.5 conditions. (**H**) FT-IR spectra of zein, pectin, free ART, and Zein_NP@ART, verifying the chemical interactions among the components. (**I-J**) Long-term stability of nanoparticles stored at 4 °C for up to 10 weeks. (**I**) Dynamic changes in particle size (left y-axis, nm) and zeta potential (right y-axis, mV) of Zein_NP and Zein_NP@ART during storage. (**J**) Polydispersity index (PDI) of Zein_NP and Zein_NP@ART over the storage period, reflecting the temporal stability of their particle size distribution uniformity. (**K**) Hemolysis percentage of erythrocytes treated with Zein_NP, Zein_NP@ART, or free ART (as control) at concentrations ranging from 25 to 2,000 μg/mL following incubation at 37 °C for 2 h, evaluating the blood compatibility of the nanocarriers. (**L**) Cytotoxicity of Zein_NP and Zein_NP@ART on HepG2 cell viability, determined by a cell counting kit-8 (CCK-8) assay at concentrations of 25–2,000 μg/mL. Data were presented as mean ± SD from three independent experiments with technical triplicates. Statistical significance was analyzed using Student’s *t*-test, with all experimental groups compared with the control group. * *P* < 0.05, ** *P* < 0.01, ***P < 0.001.

Artemisinin, a natural active compound derived from *Artemisia annua*, is widely recognized for its potent and broad-spectrum antimalarial activity against *Plasmodium* parasites. However, its inherent poor aqueous solubility limits its bioavailability and clinical applicability. Zein, a hydrophobic plant-based protein, possesses inherent amphiphilic properties and excellent biocompatibility, making it a promising candidate for the encapsulation of hydrophobic drugs (42). As shown in Fig. 1A and B, zein can be completely solubilized in an ethanol-water mixed solvent where it unfolds to form linear molecular chains. Meanwhile, ART also achieves stable dissolution in this solvent system. The microstructure of Zein_NP@ART was characterized using scanning electron microscopy (SEM). Zein particles, prepared via the anti-solvent method, displayed a spherical shape with noticeable variability in size (Fig. 1C). In contrast, free ART aggregated into block-like crystalline structures after dissolution and drying (Fig. 1D). Notably, in the SEM images of Zein_NP@ART, no separate block-like ART crystals were observed, suggesting that the engineered nanocarrier has efficiently encapsulated ART within their structure, achieving a high encapsulation efficiency (Fig. 1E and F). Given that both zein and ART are hydrophobic, ART can be encapsulated within the hydrophobic domains of unfolded zein molecular chains during the self-assembly process. However, zein exhibits an isoelectric point at a pH of 6.5, whereas at this pH, the protein carries a near-neutral net charge, leading to reduced electrostatic repulsion between particles and poor aqueous dispersibility in water (35). To address this, pectin (a negatively charged polysaccharide) was used to form a complex with zein via an electrostatic interaction to improve the dispersibility of pure zein particles. As shown in Fig. 1G, pure zein particles tend to aggregate and fail to disperse uniformly in neutral aqueous solutions. However, those pectin-modified nanoparticles markedly enhanced dispersibility across both acidic and neutral aqueous systems. The formation of these composite particles was further validated by infrared spectroscopic (IR) analysis, as illustrated in Fig. 1H. The IR spectrum of the composite formulation shows the characteristic absorption peak of ART at 1,731 cm^−1^. Additionally, a notable shift in the hydroxyl stretching vibration peak of zein was observed, which collectively confirms the successful fabrication of the zein-based sustained release formulation.

Long-term storage stability of nanocarrier formulations is a critical factor due to the fact that poor stability (e.g., particle aggregation, size variation, or structural degradation) can directly compromise drug encapsulation efficiency, *in vivo* pharmacokinetic behavior, and ultimately therapeutic efficacy (43). Additionally, the isoelectric point of the composite shifts from 6.5 to below 2, while the particle size exhibits minimal variation across various pH levels (Fig. S1A). Increasing the amount of pectin used leads to smaller composite particles with higher zeta potential, thereby enhancing the dispersibility of zein nanoparticles (Fig. S1B). Then, the storage stability of Zein_NP@ART nanoformulation was systematically evaluated by monitoring three key physicochemical parameters, including particle size distribution, polydispersity index (PDI), and zeta potential over 10 weeks. As shown in Fig. 1I, the Zein_NP@ART formulation maintained a relatively uniform particle size distribution over at least 6 weeks of storage, and no significant changes in the average particle size of the nanoparticles were detected via dynamic light scattering (DLS) analysis. Meanwhile, the zeta potential of the Zein_NP@ART formulation remained within a stable range, with no obvious fluctuations observed during the 8-week observation period. The slight increase in particle size could be partly due to shielding of the charged surfaces by the ionic strength of phosphate-buffered saline (PBS) (28). Additionally, the PDI of Zein_NP@ART remained at a low level (< 0.3) throughout an 8-week storage period (Fig. 1J). No significant fluctuations or upward/downward trends in PDI were detected during this extended storage duration. Simultaneously, the stability of zein nanoparticles was compared with that of two other common carriers, including poly(lactide-co-glycolide) (PLGA) nanoparticles and liposome (LIPO) nanoparticles. As shown in Fig. S1C, D, G, and H, both PLGA_NP@ART and LIPO_NP@ART exhibited stability changes during storage, in which their particle sizes increased slightly, zeta potential showed a gradual upward trend, and PDI rose continuously to a final value exceeding 0.3. Mechanistically, increased particle size may result from nanoparticle aggregation, reduced zeta potential absolute value indicates weakened electrostatic repulsion, and elevated PDI reflects a more heterogeneous particle size distribution. Collectively, these results confirm that both PLGA_NP@ART and LIPO_NP@ART have relatively poor storage stability, which is inferior to that of zein nanoparticles. However, this stability performance may be associated with the unmodified surface of the two carriers rather than an inherent limitation of the carrier materials themselves, as surface modification is a common strategy to enhance nanoparticle stability (44).

Biocompatibility is a key factor for nanoformulations, necessitating the investigation of both hemolytic effects and cytotoxicity. Given that ART has a potential adverse effect on erythrocytes, the hemolytic activity of free ART, Zein_NP, and Zein_NP@ART was evaluated via standard hemolysis assays. As shown in Fig. 1K, free ART showed a concentration-dependent increase in hemolytic activity, with obvious erythrocyte damage observed around 100 μg/mL. In contrast, Zein_NP@ART exhibited markedly reduced hemolytic activity across the entire concentration range. Even at the highest concentration of 2,000 μg/mL, the hemolysis rate of Zein_NP@ART remained <5% and was comparable to that of Zein_NP. This result confirms that encapsulating ART in zein nanoparticles effectively reduces the inherent hemotoxicity of free ART, which is associated with the zein-pectin composite shell on the nanoparticle surface. Cell viability was quantified via CCK-8 in which HepG_2_ cells were incubated with different concentrations of the formulations. Similarly, for Zein_NP@ART, cell viability was maintained at a high level comparable to that of Zein_NP, indicating that the encapsulated formulation retains low cytotoxicity while delivering ART (Fig. 1L). Similarly, the biocompatibility for both PLGA_NP@ART and LIPO_NP@ART was also investigated. In both sets of results, as the concentration of PLGA_NP@ART or LIPO_NP@ART increased (up to 2,000 μg/mL), the hemolysis rate remained at a low level, showing no significant upward trend (Fig. S1E and I). Meanwhile, the cell viability results of PLGA_NP@ART and LIPO_NP@ART were comparable to those of Zein_NP@ART, confirming that these two nanocarrier formulations also possess good biocompatibility (Fig. S1F and J). These results indicate that, like zein nanoparticles, both PLGA_NP@ART and LIPO_NP@ART exhibit lower cytotoxicity and can effectively reduce the inherent hemolytic activity of free ART.

**Fig 2:**
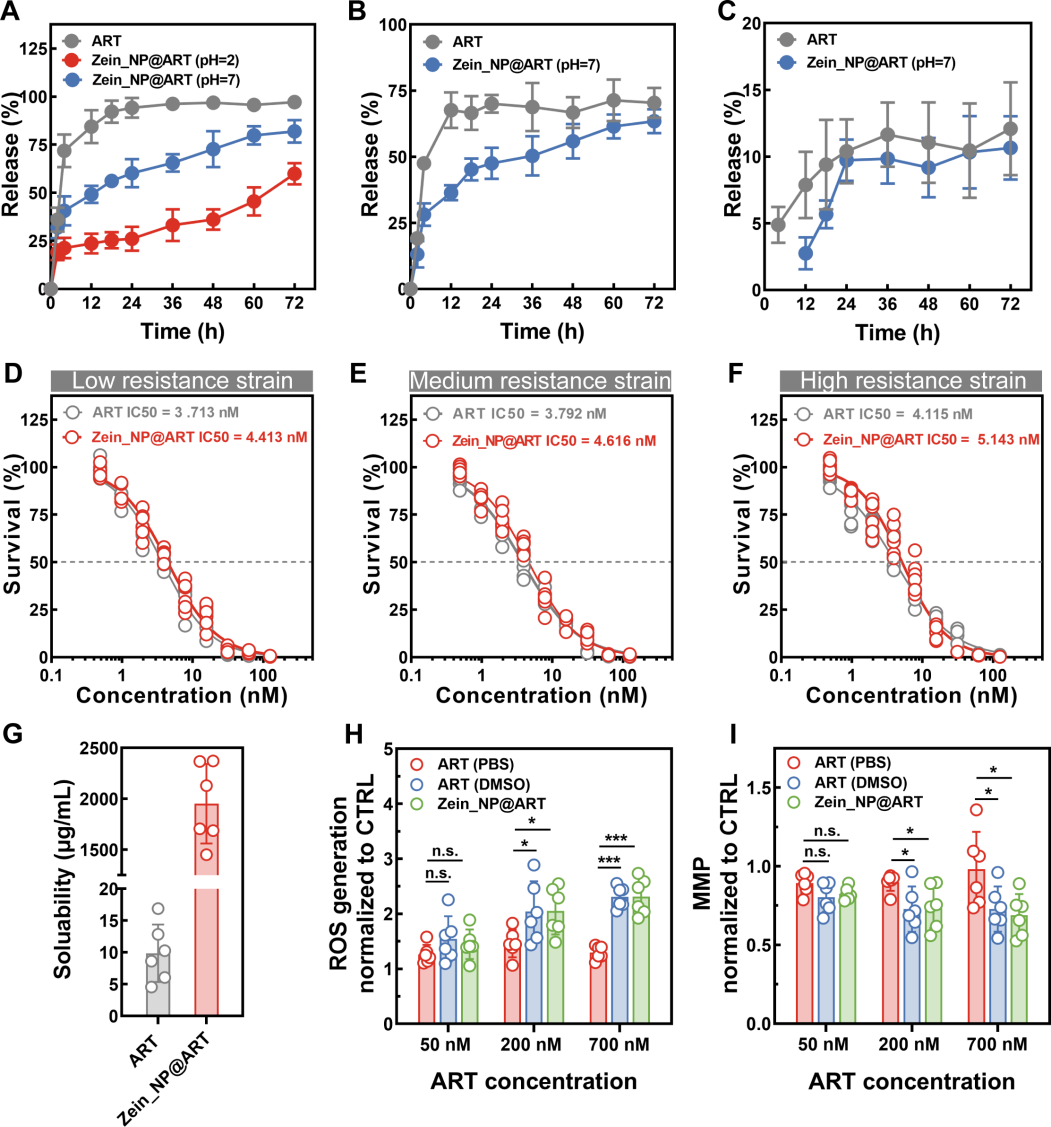
*In vitro* antimalarial activity for Zein_NP@ART due to sustained release profile and enhanced solubility. (**A**) Cumulative release profiles of ART from Zein_NP@ART under varying pH conditions, with free ART being set as a control. Data were presented as mean ± SD from three independent experiments with technical triplicates. (**B-C**) To investigate in detail the ART concentration entering into the RBCs, a mimic buffer system containing RBCs (at 50% hematocrit) and supplemented with Albumax was used. At predetermined time points, samples were collected and centrifuged, and RBCs were lysed using a Pall filter for quantification of intracellular ART. Data were presented as mean ± SD from two independent experiments with technical triplicates. To obtain cumulative release profiles, the measured ART concentrations (in both supernatant and RBCs) were normalized to the initial ART concentration. (**D–F**) Typical dose-response evaluations of free ART and Zein_NP@ART on asynchronous cultures of *P. falciparum* strains with different levels of ART resistance during the asexual stage. A 3-day SYBR Green I-based dose-response assay was performed to quantify parasite growth. The parasites were exposed to a series of concentrations for 72 h (with an [inhibitor] vs. normalized response fit). These analyses were performed using nonlinear regression in GraphPad Prism 8.0 software. (**G**) The solubility of free ART and ART encapsulated in Zein_NP@ART in water. Significantly enhanced solubility of ART was achieved in Zein_NP@ART, which was attributed to the inherent properties of Zein_NP. (**H-I**) Effects of Zein_NP@ART on reactive oxygen species (ROS) generation and mitochondrial function. Given the enhanced solubility of ART in Zein_NP@ART, the antimalarial activity of the prepared Zein_NP@ART was evaluated by comparing its biological effects with those of ART in either PBS or DMSO. All results were normalized to the values obtained from the vehicle control group. Data were presented as mean ± SD from two independent experiments with technical triplicates. Statistical significance was analyzed using one-way analysis of variance (ANOVA) followed by multiple comparison tests with Bonferroni correction. * *P* < 0.05, ** *P* < 0.01, ***P < 0.001.

Zein_NP@ART showed different dispersibility and zeta potential across different pH environments, indicating its versatility and utility in both acidic and neutral settings. This paves the way for its broad use in both oral and intravenous deliveries. Figure 2A depicts the release profiles of free ART in water compared to ART encapsulated in nanoparticles, highlighting different release patterns at acidic and neutral pH levels. Both curves of Zein_NP@ART demonstrate obvious sustained release effects, which differ significantly from that of free ART, which was almost completely released within the first 12 h. Specifically, results showed that the half-life of free ART is around 3 h, indicating that it needs frequent and significant dosages to achieve therapeutic benefits. Additionally, significant differences in release kinetics were observed for Zein_NP@ART between the two pH conditions. At pH 7, Zein_NP@ART showed a relatively fast drug release rate: the cumulative release amount increased rapidly within the initial 24 h, and the cumulative release rate reached approximately 75% by 72 h. At pH 2, the formulation exhibited a more prolonged sustained-release characteristic, and the drug release rate was notably slower, with the cumulative release amount within the first 24 h being significantly lower than that observed at pH 7. Thus, compared with free ART, integrating ART into Zein_NP markedly extends its half-life to 12 h in neutral conditions and 67 h in acidic environments. Additionally, the *in vitro* release profile of PLGA_NP@ART and LIPO_NP@ART has also been investigated. As shown in Fig. S2A, the cumulative ART release remained relatively low under both conditions over the 72 h detection period, likely due to the slow degradation of PLGA’s polymer matrix that restricts drug diffusion. In contrast, a distinct burst release of ART was observed in LIPO_NP@ART, possibly attributed to the destabilization of the liposomal bilayer under acidic conditions that accelerates rapid drug leakage (Fig. S2B). Thus, either the insufficient or burst release may compromise its *in vivo* antimalarial activity and circulation period.

To further clarify the actual amount of ART that enters erythrocytes, another *in vitro* release experiment was conducted using a simulated blood buffer containing erythrocytes and albumin. Similarly, a sustained-release profile was observed for Zein_NP@ART in which ART was released from Zein_NP@ART slowly and continuously into the extracellular fluid, without a burst release phenomenon (Fig. 2B). For both free ART and Zein_NP@ART, the intracellular ART content in erythrocytes increased gradually in the early stage of incubation and reached a stable equilibrium at approximately 24 h (Fig. 2C). No significant changes in intracellular ART content were detected after 24 h, confirming that Zein_NP@ART enables efficient and timely delivery of ART into erythrocytes.

To investigate whether encapsulating ART into nanocarriers affects its antimalarial activity, drug sensitivity assays were performed against drug-sensitive (Fig. S2C) and ART-resistant parasite strains (Fig. 2D through F). Compared to free ART, the half-maximal inhibitory concentration (IC₅₀) of ART slightly increased after encapsulation. This slight elevation was consistent across the drug-sensitive strain and low, medium, and high resistance strains. Notably, this slight IC₅₀ increase did not compromise the overall antimalarial activity of ART, confirming that ART’s chemical properties and effectiveness remain stable within the nanoformulation. Consistent with the *in vitro* release profiles, PLGA_NP@ART exhibited a slight increase in IC₅₀ values compared to Zein_NP@ART when tested against both the WT and ART-resistant parasite strains (Fig. S2F through I). In contrast, LIPO_NP@ART showed significantly higher IC₅₀ values than Zein_NP@ART across the same strains (Fig. S2F through I). This notable difference is attributed to the insufficient ART release from LIPO_NP under neutral conditions.

Notably, the poor aqueous solubility and dispersibility of free ART have long been key factors restricting its bioavailability, therapeutic efficacy, and clinical application scope. As demonstrated in Fig. 2G, following incorporation into Zein_NP@ART, the aqueous solubility of ART was significantly enhanced from 10 µg/mL to 2,000 µg/mL, representing a 200-fold improvement. Similarly, PLGA_NP@ART and LIPO_NP@ART both exhibited significantly enhanced water solubility compared to free ART, with the solubility of PLGA_NP@ART increased by ~250-fold and that of LIPO_NP@ART improved by ~40-fold (Fig. S2D and E). These results clearly demonstrate the varying degrees of solubility enhancement achieved by different nanoparticle delivery systems and further confirm the feasibility of encapsulating ART into nanoparticles to address its poor aqueous solubility issue.

Since ART-based drugs primarily exert their antimalarial effects by generating ROS to disrupt the parasite’s mitochondrial function (45, 46), we investigated how the nanoformulation affects this mechanism. Based on the enhanced aqueous solubility of ART achieved via zein nanoparticle encapsulation, we further investigated the effects of different ART formulations on ROS production and mitochondrial function across varying concentrations, with ART dissolved in DMSO serving as the positive control (Fig. 2H and I). The results showed that at 50 nM, Zein_NP@ART exhibited no significant difference from free ART in aqueous solution in terms of ROS generation and regulation of mitochondrial function. In contrast, at high concentrations (200 nM and 700 nM), Zein_NP@ART induced notably higher ROS production and exerted more potent inhibitory effects on mitochondrial function. This concentration-dependent advantage is directly linked to the improved aqueous solubility of ART in the nanoformulation.

**Fig 3:**
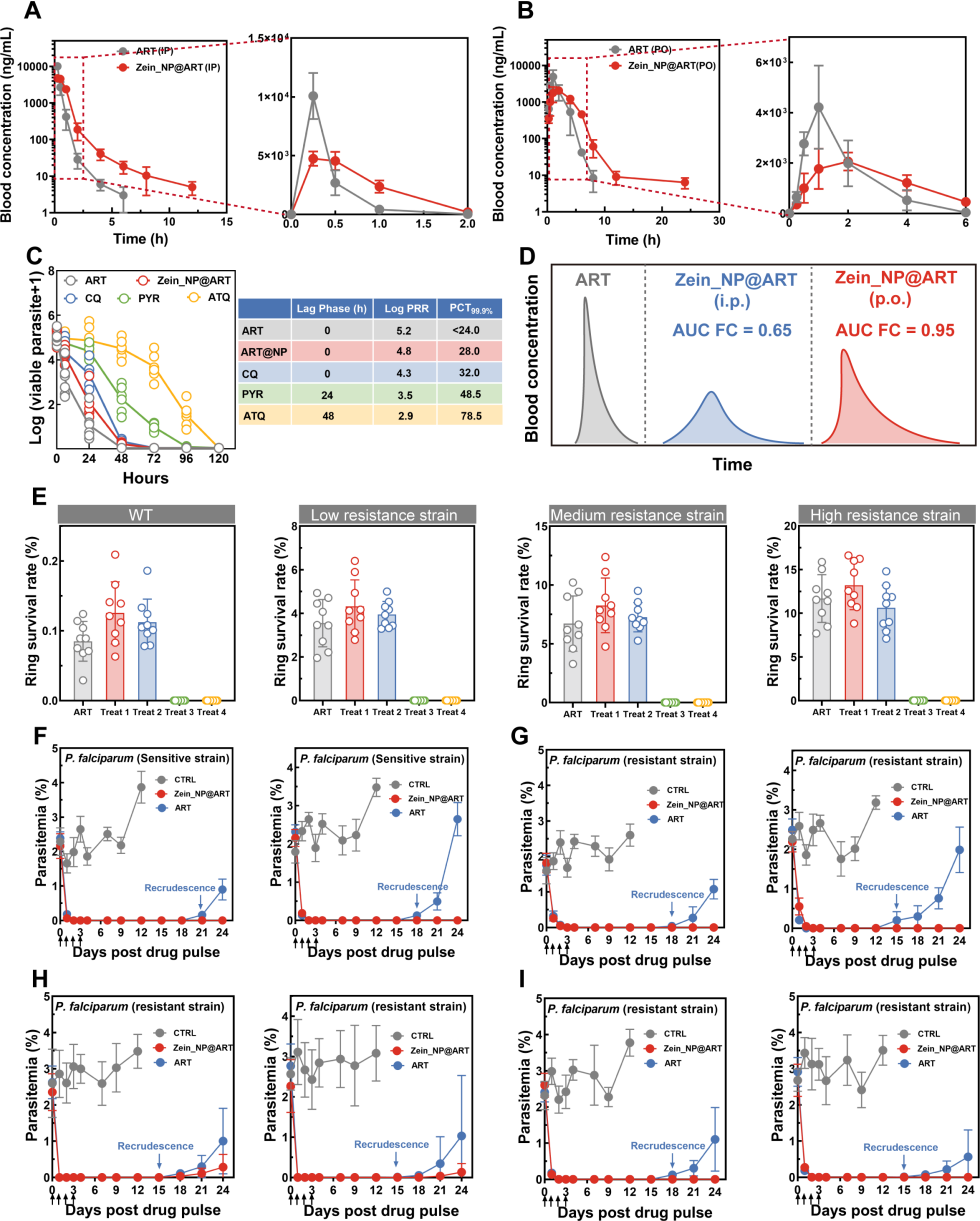
Enhanced *in vitro* antimalarial activity of Zein_NP@ART against ART-resistant parasite strains mediated by prolonged half-life. (**A-B**) Pharmacokinetic profiles of ART and Zein_NP@ART following intravenous and oral administration at a dosage of 50 mg/kg; the inset shows blood drug concentrations in the early stage after administration. Data were presented as mean ± SD from 3 mice for each time point. (**C**) *In vitro* PRR assay was performed to evaluate the mode of action (MOA) of Zein_NP@ART and traditional antimalarials (chloroquine, pyrimethamine, and atovaquone); the key parameters obtained from this assay are listed in the table. Data were presented as mean ± SD from two independent experiments with technical triplicates. (**D**) Schematic illustration of the selection basis for ART exposure concentration and duration in phenotypic assays against ART-resistant parasite strains. (**E**) Ring survival rate for parasites with varied resistance obtained in modified RSA based on the pharmacokinetic profile for Zein_NP@ART. Treat 1: parasites exposed to 450 nM ART for 6 h. Treat 2: parasites exposed to 650 nM ART for 6 h. Treat 3: parasites exposed to 450 nM ART for 20 h. Treat 4: parasites exposed to 650 nM ART for 22 h. Data were presented as mean ± SD from three independent experiments with technical triplicates. Statistical significance was analyzed using Student’s *t*-test, with all experimental groups compared with the control group. * *P* < 0.05, ** *P* < 0.01, ***P < 0.001. (**F–I**) Recrudescence curves of *P. falciparum* parasites exposed to four daily pulses with different ART formulations. WT (**F**), low resistance (**G**), medium resistance (**H**), and high resistance (**I**) parasite strains were subjected to different ART pulses (left panel: 450 nM for 20 h, right panel: 650 nM for 22 h) and the parasitemia profile was recorded at the corresponding time point. The vehicle control cultures were diluted every 48 h and monitored for at least 10 days. Black arrows indicate the time point of the ART pulse, and blue arrows correspond to parasite recrudescence. Data were presented as mean ± SD from two independent experiments with technical triplicates.

Given that free ART has a short *in vivo* half-life, which limits its therapeutic duration, we further investigated whether zein nanoparticles could improve this pharmacokinetic property by evaluating the blood concentration-time profiles of ART and Zein_NP@ART. The pharmacokinetic profiles for the formulation by either intraperitoneal or oral administration at a dosage of 50 mg/kg are shown in Fig. 3A and B, and the corresponding pharmacokinetic parameters are given in Table 1 and the Source Data file. For free ART, the blood concentration peaked rapidly in the early phase and then declined sharply via both administration routes. In contrast, Zein_NP@ART avoided this rapid fluctuation, in which the enlarged early-phase data revealed that Zein_NP@ART maintained more stable initial blood concentrations, and an extended rapid distribution phase followed by a prolonged terminal elimination phase was observed, which was correlated with the previous *in vitro* release assay without a burst of drug release. As shown in Table 1, free ART exhibits rapid systemic clearance *in vivo*, with clearance rates (CL) of 0.57 L/h/kg following intraperitoneal administration and 0.83 L/h/kg after oral administration, respectively. Consistent with this rapid clearance profile, free ART also demonstrates a short half-life (t₁/₂) of 0.91 h and 1.20 h, as well as a relatively short mean residence time (MRT) of 0.18 h and 1.68 h. The rapid clearance of free ART is characterized by the efficient elimination of the drug from the systemic circulation and reflects its rapid metabolism and excretion in the body, a key pharmacokinetic feature of ART. While entrapped in the nanoparticle, either a reduced clearance rate or prolonged half-life with higher abundance was achieved for ART. Specifically, encapsulation results in a 4.5-fold and 1.8-fold increase in mean residence time. In contrast to free ART, which was rapidly cleared from blood plasma 6 h post-administration, Zein_NP@ART displayed a remarkable improvement in the circulation profile for either oral or intravenous administration, in which the plasma concentration gradually decreased over time, but ART remained detectable in all mice after 6 h. We further compared the pharmacokinetic profiles of PLGA_NP@ART and LIPO_NP@ART following intraperitoneal administration. As presented in Fig. S3, distinct differences in t₁/₂ were observed between the two formulations: PLGA_NP@ART exhibited a mean t₁/₂ of 1.97 ± 0.55 h, while LIPO_NP@ART showed a notably longer mean t₁/₂ of 3.56 ± 1.58 h. Importantly, both nanoformulations effectively prolonged the *in vivo* half-life of ART compared to free ART, confirming the utility of nanocarrier-based delivery in improving ART’s pharmacokinetic properties.

**TABLE 1:** Pharmacokinetic parameters for free ART and corresponding formulations

Formulation	ART solution (ip)	ART solution (po)	Zein_NP@ART (ip)	Zein_NP@ART (po)
Dose (mg/kg)	50	50	50	50
AUC0-last (h* ng/mL)	8,840 ± 754	9.015 ± 974	5,760 ± 500	8,574 ± 1,618
CL (L/h/kg)	0.57 ± 0.04	0.83 ± 0.07	0.87 ± 0.07	0.98 ± 0.09
t1/2 (h)	0.91 ± 0.04	1.20 ± 0.02	3.17 ± 0.79	4.57 ± 1.87
MRT0-last (h)	0.18 ± 0.02	1.68 ± 0.04	0.82 ± 0.13	3.18 ± 0.11

In order to investigate the mode of action of the Zein_NP@ART, especially whether nanoformulation affects ART’s rapid killing characteristic, we performed an *in vitro* parasite reduction ratio (PRR) assay to quantify the parasite killing rate of the nanoformulation and time-dependent parasite viability profile is shown in Fig. 3C. As a fast-acting antimalarial, free ART rapidly reduced the number of viable parasites to <0.1% within 48 h with a log (PRR) value higher than 5. It is followed, in decreasing speed-of-action order, by the nanoformulation, chloroquine, pyronaridine, and atovaquone with PRR values of 4.8, 4.3, 3.5, and 2.9, respectively. Thus, both free ART and the nanoformulation showed a fast-killing profile with the ability to decrease the parasitemia by ~5 orders of magnitude. The number of viable parasites was significantly decreased by 4 log units for all tested compounds within 120 h. Meanwhile, as shown in the inserted Table (Fig. 3C), there was a slightly increased PCT along with an unchanged lag phase for the nanoformulation compared to free ART, further indicating that the entrapment of ART did not affect antimalarial activity.

To verify if the developed nanoformulation could effectively target drug-resistant parasites through prolonged drug release, an extended ring-stage survival assay (RSA) was carried out. Briefly, we slightly modified the standard RSA according to its core experimental principles (47). Based on the previously obtained pharmacokinetic parameter (i.e., AUC_0-last_ and t_1/2_) of the nanoformulation, we calculated the equivalent exposure dosage, which was normalized to match the standard ART exposure conditions (700 nM concentration and 6 h duration) to ensure comparability of the RSA results (Fig. 3D). As shown in Fig. 3E, when tested under the standard 6-h exposure condition with the calculated equivalent exposure dosages (450 nM and 650 nM), the therapeutic effectiveness of nanoformulation was slightly lower than that of free ART, which is presumably attributed to the incomplete release of ART from the zein nanocarriers within the 6 h. However, when the assay conditions were optimized to match real-life pharmacokinetic exposure, the results clearly demonstrated that employing Zein_NP as a sustained-release carrier significantly reduced the ring survival rate. Against drug-resistant *Plasmodium* strains, parasites treated with Zein_NP@ART exhibited markedly enhanced antimalarial efficacy compared to those exposed to free ART under standard concentration and treatment duration, with no viable parasites detected post-treatment.

To assess the ability of the proposed Zein_NP@ART nanoformulation to inhibit recrudescence for both sensitive and resistant strains, we conducted *in vitro* recrudescence assays designed using pharmacokinetic parameters calculated above. Unsynchronized parasites were subjected to 4 consecutive days of drug pulse, a period equivalent to two complete asexual cycles. The recrudescence curve was plotted based on parasitemia and is shown in Fig. 3F through I. We observed that the parasitemia in each of these experiments rapidly decreased to a low level after day 2. Parasites in the DMSO vehicle control group grew normally and rose to above 2% parasitemia. Free ART and Zein_NP@ART differed sharply in recrudescence inhibition. Specifically, in cultures exposed to free ART, living parasites were observed for both sensitive and resistant strains. In contrast, no detectable living parasites were observed for the Zein_NP@ART treated culture over the same period. This superiority confirms that Zein_NP@ART could effectively suppress parasite recrudescence, strongly indicating its potential to improve ART resistance.

**Fig 4:**
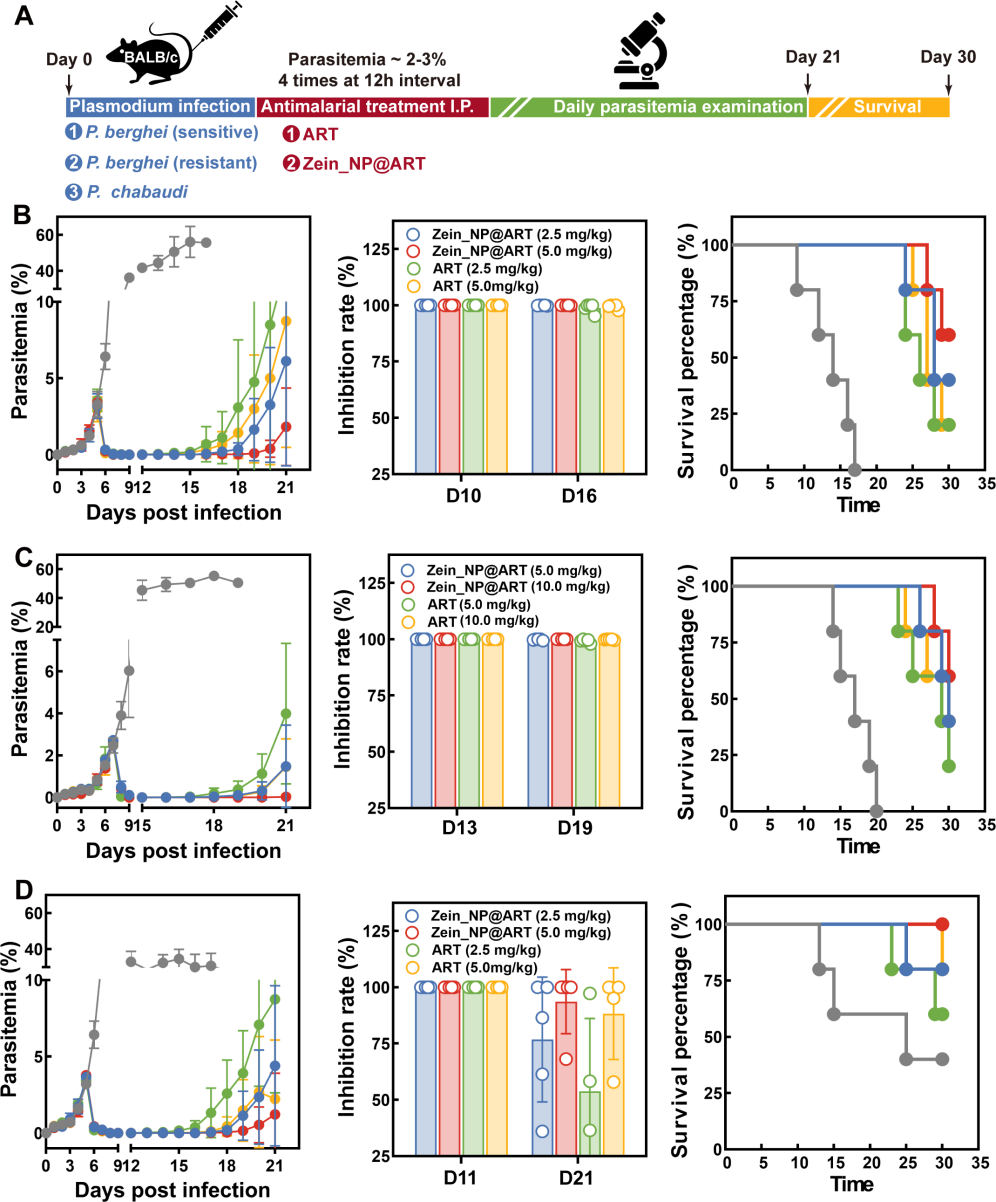
*In vivo* antimalarial activity evaluation of intraperitoneal Zein_NP@ART treatment in a rodent model with a 12 h interval. (**A**) Schematic illustration of parasite infection and Zein_NP@ART treatment: Female mice were intraperitoneally infected with 10⁶ parasites on Day 0. Antimalarial intervention was initiated on Day 4 at 12 h intervals, by intraperitoneal injection of corresponding ART formulations when peripheral blood parasitemia had reached 2%–3%. Parasitemia of *P. berghei* WT strain (**B**, left panel), *P. berghei*-resistant strain (**C**, left panel), and *P. chabaudi* (**D**, left panel) was determined in five experimental groups: vehicle control group, low-dose free ART group (2.5 mg/kg), medium-dose free ART group (5.0 mg/kg), and Zein_NP@ART-treated group with an equal amount of ART. Parasitemia levels were quantified daily by an experienced technician via microscopic examination of Giemsa-stained blood smears from at least 5,000 RBCs. Data were presented as mean ± SD with 5 mice in each group. Parasite inhibition rate for *P. berghei* WT strain (**B**, middle panel), *P. berghei*-resistant strain (**C**, middle panel), and *P. chabaudi* (**D**, middle panel) infected mice administrated with different formulations of ART at corresponding time point (Due to inherent differences in parasite developmental stages and experimental endpoint definitions across groups, detailed time points for inhibition rate assessment are provided in the SourceData file). Kaplan-Meier survival curve for all groups of mice post-infection of *P. berghei* WT strain (**B**, right panel), *P. berghei*-resistant strain (**C**, right panel), and *P. chabaudi* (**D**, right panel). Survival outcomes of mice in all experimental groups were monitored continuously post-infection.

The *in vivo* antimalarial activity for the corresponding nanoformulation was initially evaluated in rodent malaria parasites, in which the efficiency was assessed with different parasite strains. The detailed experimental design was illustrated in Fig. 4A. For mice infected with the drug-sensitive strain of *P. berghei*, distinct antimalarial effects were observed post-drug administration. Both the free ART group and the Zein_NP@ART group induced a significant reduction in parasitemia levels. Meanwhile, on the pre-designated observation days, both treatment groups exhibited favorable and dose-dependent parasite inhibition rates, which is likely attributed to the potent intrinsic antimalarial activity of ART. Notably, over the 30-day survival observation period, the Zein_NP@ART group demonstrated improved cumulative survival rates (60% survival at Day 30) compared to the free ART group (20% survival at Day 30) (Fig. 4B). This finding suggests that zein nanoparticle encapsulation not only preserves the inherent antimalarial activity of ART but also enhances its long-term protective efficacy in infected mice. Similarly, in mice infected with the ART-resistant *P. berghei* strain, a comparable trend to that of the sensitive strain was observed in which the Zein_NP@ART group still outperformed the free ART group in overall therapeutic efficacy. Specifically, Zein_NP@ART treatment led to marked reductions in parasite recrudescence rates and a concurrent improvement in long-term survival outcomes (Fig. 4C). In addition to *P. berghei*, another rodent model infected by *P. chabaudi* was used to evaluate the protective effect on chronic infection. Critically, the chronic infection phenotype of this murine model enables it to faithfully mimic the “relapse and recrudescence” phenomena that are clinically relevant to human malaria, addressing a key limitation of models with acute, non-recurrent infection profiles. Results demonstrate that, in contrast to the *P. berghei* model, the high-dose Zein_NP@ART exhibits enhanced antiparasitic efficacy relative to the free drug group. Zein_NP@ART not only outperforms the free drug in suppressing parasite proliferation but also markedly elevates the parasite inhibition rate, as quantified by parasitemia monitoring (Fig. 4D).

**Fig 5:**
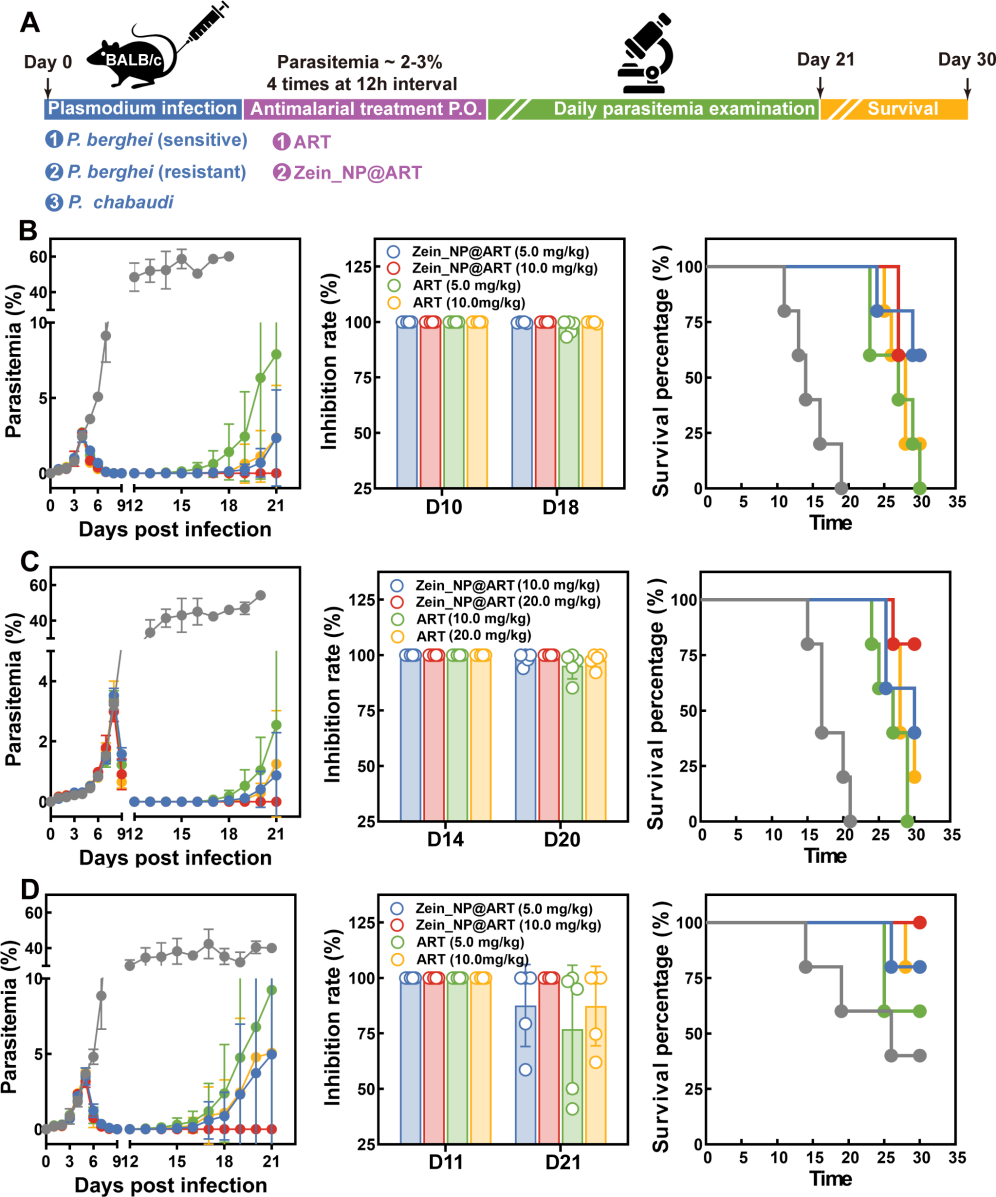
*In vivo* antimalarial activity evaluation of oral Zein_NP@ART treatment in rodent model with a 12 h interval. (**A**) Schematic illustration of parasite infection and Zein_NP@ART treatment: Female mice were intraperitoneally infected with 10⁶ parasites on Day 0. Antimalarial intervention was initiated on Day 4 at 12 h interval, by oral administration of corresponding ART formulations when peripheral blood parasitemia had reached 2%–3%. Parasitemia of *P. berghei* WT strain (**B**, left panel), *P. berghei*-resistant strain (**C**, left panel), and *P. chabaudi* (**D**, left panel) were determined in five experimental groups: vehicle control group, low-dose free ART group (5.0 mg/kg), medium-dose free ART group (10.0 mg/kg), and Zein_NP@ART-treated group with an equal amount of ART. Parasitemia levels were quantified daily by an experienced technician via microscopic examination of Giemsa-stained blood smears from at least 5,000 RBCs. Data were presented as mean ± SD with 5 mice in each group. Parasite inhibition rate for *P. berghei* WT strain (**B**, middle panel), *P. berghei*-resistant strain (**C**, middle panel), and *P. chabaudi* (**D**, middle panel)-infected mice administrated with different formulations of ART at corresponding time points (Due to inherent differences in parasite developmental stages and experimental endpoint definitions across groups, detailed time points for inhibition rate assessment are provided in the SourceData file). Kaplan-Meier survival curve for all groups of mice post-infection of *P. berghei* WT strain (**B**, right panel), *P. berghei*-resistant strain (**C**, right panel), and *P. chabaudi* (**D**, right panel). Survival outcomes of mice in all experimental groups were monitored continuously post-infection.

Previous results have shown that Zein_NP@ART could significantly enhance its aqueous solubility, laying a foundation for expanding ART’s administration routes. Then, we have further validated the *in vivo* antimalarial activity of Zein_NP@ART via oral administration. Given that oral drug bioavailability is markedly lower than that of intraperitoneal injection, the ART dosage in the oral group was adjusted to ensure comparable drug exposure in the bloodstream between oral and intraperitoneal groups (Fig. 5A). Oral administration exhibited antimalarial efficacy consistent with intraperitoneal injection. Relative to the oral free ART group, the Zein_NP@ART treated group effectively reduced parasite recrudescence (3–5 days delayed recrudescence in *P. berghei*-sensitive/resistant strains and *P. chabaudi* models, respectively) and significantly improved survival of infected mice (Fig. 5B through D). Notably, in all three infection models, no parasite recrudescence was detected in the high-dose oral Zein_NP@ART treated group, with parasitemia remaining below the detection limit until the observation endpoint. This confirms that nanocarrier formulations boost ART’s oral absorption efficiency and prolong intestinal drug retention, thereby enhancing ART’s antimalarial activity via oral delivery, providing experimental support for the clinical development of oral antimalarial nanoformulations.

According to the pharmacokinetic data, we have also investigated the impact of drug administration intervals on antimalarial efficacy. Results showed that the 24-h interval dosing group shared an overall therapeutic trend with the 12-h interval group (Fig. S4 and S5). Both groups achieved initial parasitemia reduction and extended the survival of infected mice. However, the antimalarial activity of the 24-h interval treatment was significantly less potent than that of the 12-h interval treatment. This outcome is likely due to the rapid metabolism and clearance of ART. In the 24-h group, blood drug concentrations drop below the effective parasiticidal threshold before the next dose. This creates a prolonged “drug-free window” that enables residual parasites to survive. In contrast, the 12-h interval maintains consistent therapeutic concentrations. It minimizes the drug-free window and achieves superior antimalarial efficacy.

**Fig 6:**
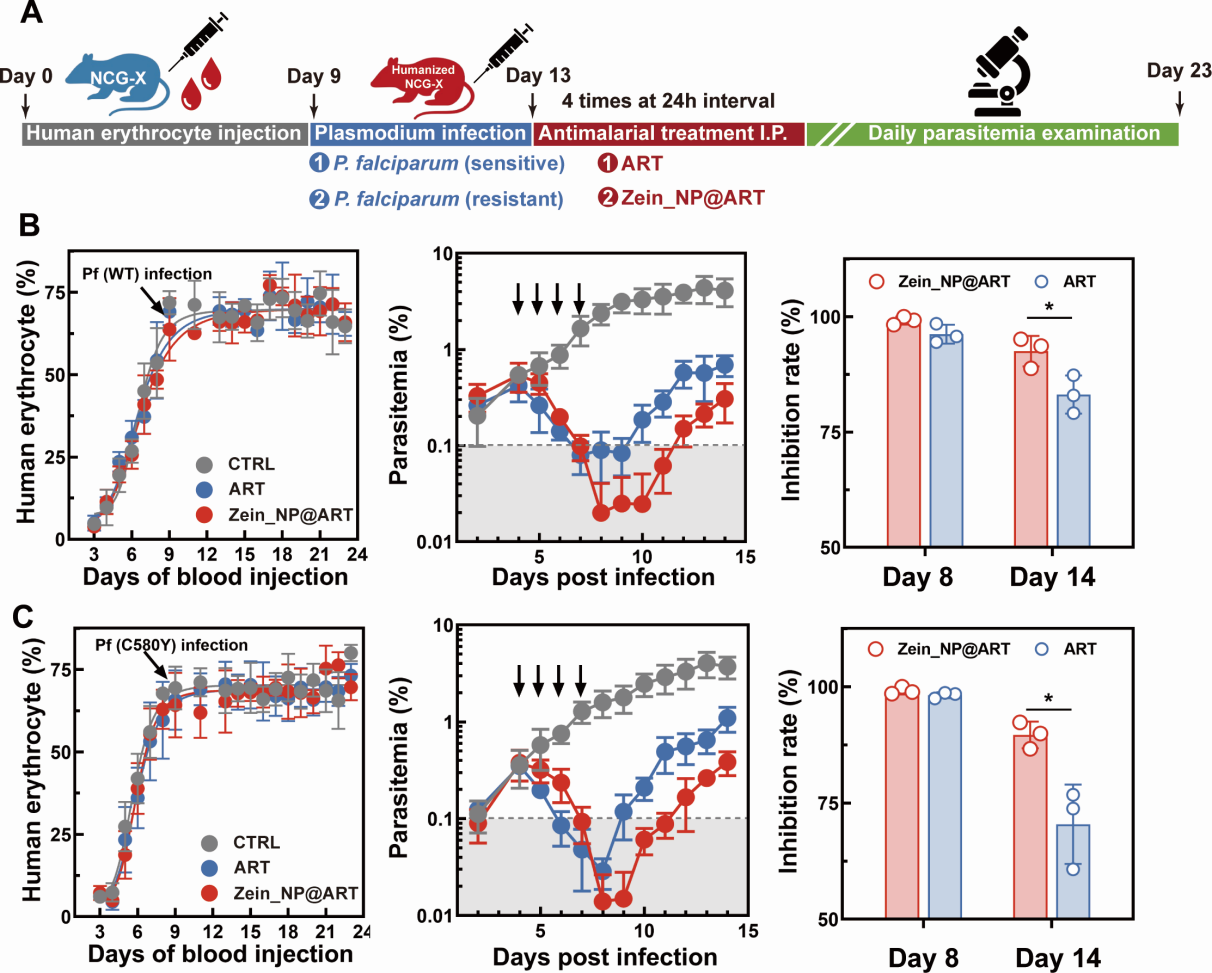
*In vivo* antimalarial efficiency for Zein_NP@ART in a humanized mice model infected with *P. falciparum*. (**A**) The schematic illustration of the design of the experiment. Female NCG-X mice were intraperitoneally engrafted with human erythrocyte suspensions via daily administration from day 0 to day 9. On day 9, each mouse received an intravenous injection of 3 × 10⁷ parasitized red blood cells (pRBCs) in a total volume of 0.1 mL for *P. falciparum* infection; 4 days post-infection, mice were treated with different formulations of ART, and parasitemia was quantified daily. Antimalarial effect of Zein-NP@ART against *P. falciparum* WT (**B**) and C580Y (**C**) strain infected NCG-X mice. Left panel: Measurement of engraftment efficiency by flow cytometry. Engraftment efficiency was quantified based on the percentage of human erythrocytes in the peripheral blood of NCG-X mice. The engraftment efficiency was measured according to the percentage of human erythrocytes by flow cytometry. Middle panel: Parasitemia was microscopically assessed based on Giemsa-stained blood smears and calculated from at least 10,000 RBCs. Right panel: Parasite inhibition rate at the corresponding time point. Three mice were used in each group. Statistical significance was analyzed using Student’s *t*-test. * *P* < 0.05.

A humanized mouse model was applied to explore the effects of corresponding nanoformulation against *P. falciparum*. This model holds unique advantages for studying *P. falciparum,* which exclusively infects human erythrocytes. It overcomes the species barrier of conventional murine models by providing a physiological environment that mimics human blood. Briefly, immune-deficient mice were engrafted with human erythrocytes daily for *P. falciparum* infection (both ART-sensitive and -resistant strains) (Fig. 6A). Parasitemia levels were dynamically monitored via Giemsa-stained blood smears throughout the experiment, serving as a critical readout for both infection validation and real-time tracking of therapeutic responses (Fig. 6B and C). The parasitemia curve results clearly demonstrated that Zein_NP@ART exerted significantly stronger lethality against *P. falciparum* (both drug-sensitive and -resistant strains) compared to free ART. Specifically, after 4 consecutive days of treatment at a dosage of 5 mg/kg, a sharp reduction in parasite proliferation was observed in Zein_NP@ART-treated mice relative to the control group. All mice receiving Zein_NP@ART survived the entire 14-day observation period post-infection, further confirming the formulation’s *in vivo* efficiency. Notably, the nanoformulation achieved an 85% parasite inhibition rate for both strains by Day 14, a performance superior to free ART, which typically shows diminished activity against resistant strains.

## DISCUSSION

According to the guidelines issued by the WHO, ART derivatives are recommended as the first-line treatment for malaria. However, the oil-based dosage form of ART is associated with erratic absorption and non-consistent pharmacokinetics, which leads to fluctuating blood drug concentrations, suboptimal therapeutic exposure, and ultimately compromised clinical efficacy. To address these critical limitations, we formulated ART into a water-soluble form using nanotechnology to enhance the activity against ART-resistant parasite strains both *in vitro* and *in vivo*. Our findings demonstrate the potential of zein-based nanoformulation (Zein_NP@ART) in enhancing the efficacy of ART for malaria treatment. By addressing critical challenges associated with ART, including poor aqueous solubility, limited oral bioavailability, rapid metabolic clearance, and reduced efficacy against drug-resistant *Plasmodium* strains, our nanoformulation offers a promising alternative to conventional antimalarial therapies. Its improved pharmacokinetic properties and broad-spectrum activity against resistant parasites align with global efforts to combat malaria and overcome the growing threat of antimalarial drug resistance.

Storage stability is critical for nanocarrier formulations as poor stability compromises drug encapsulation efficiency, pharmacokinetic behavior, and therapeutic efficacy. Systematic evaluation over 10 weeks shows Zein_NP@ART has excellent storage stability. It maintains uniform particle size distribution for at least 6 weeks with no significant average size changes. Zeta potential stays stable for 8 weeks without obvious fluctuations. A slight particle size increase may relate to PBS ionic strength shielding charged surfaces. PDI remains below 0.3 for 8 weeks with no significant fluctuations.

Comparison with PLGA_NP@ART and LIPO_NP@ART confirms Zein_NP@ART’s superior stability. The latter two show increased particle size, gradual zeta potential elevation, and PDI exceeding 0.3 during storage, which may result from aggregation, weakened electrostatic repulsion, and heterogeneous particle size distribution. Their relatively poor stability is likely associated with unmodified surfaces rather than inherent carrier limitations, as surface modification can enhance nanoparticle stability. Overall, these findings demonstrate Zein_NP@ART possesses excellent storage stability.

In clinical treatment of malaria, hemolysis is often observed after ACT, possibly mediated through the toxic oxidative effect of ART derivative on the erythrocyte membrane. Thus, in this study, the hemolytic effects of free ART and nanoformulations were investigated. The results demonstrate that free ART has concentration-dependent hemolytic activity with obvious erythrocyte damage. While encapsulation into the nanoformulation, Zein_NP@ART, significantly reduces this hemolytic activity across all concentrations, suggesting that nanoformulation mitigates ART’s inherent hemotoxicity, possibly due to the zein-pectin composite shell. Biocompatibility assessments revealed that Zein_NP@ART exhibits negligible cytotoxicity toward HepG_2_ cells, even at high concentrations. Meanwhile, both PLGA-NP@ART and LIPO_NP@ART also exhibit favorable biological safety. These findings collectively validate the feasibility of the nanocarrier-based strategy for mitigating ART-associated toxicity, providing experimental support for the rational design of safe antimalarial nanoformulations.

The sustained-release properties of Zein_NP@ART play a pivotal role in overcoming the issue of parasite dormancy and reducing the likelihood of resistance development. It significantly prolongs ART’s half-life, achieving continuous release under both acidic and neutral conditions without burst release. In contrast, PLGA_NP@ART shows insufficient drug release, and LIPO_NP@ART exhibits burst release, which may impair *in vivo* efficacy. Even in a simulated blood environment, Zein_NP@ART maintains stable, sustained release and efficiently delivers ART to erythrocytes, which ensures a continuous therapeutic presence in the bloodstream. This extended release not only maintains drug concentrations above the IC_50_ threshold for an extended period but also minimizes the need for frequent dosing, thereby enhancing patient compliance and reducing the risk of adverse effects associated with higher doses.

One of the primary limitations of ART is its poor water solubility, which significantly restricts its bioavailability and therapeutic applicability. Our results indicate that encapsulating ART within zein nanoparticles maintained its stability in acidic and alkaline conditions and increased ART’s aqueous solubility by 200-fold, a substantial improvement that could lead to more consistent and effective drug concentrations in the target tissues. Also, these dispersibility improvements were comparable to those of PLGA_NP@ART and LIPO_NP@ART. This enhancement is critical for ensuring that sufficient drug levels are maintained to exert antimalarial effects, particularly in the challenging intracellular environment where *Plasmodium* parasites reside. This enhanced solubility supports ART’s antimalarial mechanism by generating ROS and disrupting mitochondrial function to kill parasites. It can be seen that at higher concentrations (200 nM and 700 nM), the nanoformulation induces greater ROS generation and more potent mitochondrial damage, which can be directly attributed to its improved aqueous solubility.

Drug sensitivity assays against ART-sensitive and -resistant Plasmodium strains confirmed that encapsulating ART into nanocarriers does not compromise its core antimalarial activity. Specifically, Zein_NP@ART exhibited a slight increase in IC₅₀ compared to free ART, a trend consistent across sensitive and low-to-high resistant strains without impairing overall efficacy. Additionally, PLGA_NP@ART showed slightly higher IC₅₀ values than Zein_NP@ART against both wild-type and resistant strains, while LIPO_NP@ART displayed significantly elevated IC₅₀ values relative to Zein_NP@ART, an outcome attributed to insufficient ART release under neutral conditions. Collectively, these results validated that ART’s chemical properties and parasiticidal activity remain stable and unaffected by carrier interactions.

Pharmacokinetic analysis further corroborates these findings, revealing that Zein_NP@ART exhibits a significantly prolonged *in vivo* half-life alongside increased mean residence time (MRT) and reduced systemic clearance rates compared to free ART. This pharmacokinetic improvement is consistent across both intravenous and oral administration routes, translating to delayed drug elimination and sustained plasma concentrations over an extended period. The delayed elimination and prolonged plasma retention observed in both intravenous and oral administration routes suggest a delayed drug elimination and sustained plasma concentrations over an extended period. By maintaining effective therapeutic drug levels in circulation for longer, Zein_NP@ART addresses the limitation of free ART’s rapid clearance, an advantage critical for achieving complete eradication of both sensitive and resistant Plasmodium parasites, as well as preventing post-treatment recrudescence that often arises from insufficient drug exposure.

To clarify whether Zein_NP@ART affects ART’s rapid killing property, an *in vitro* parasite reduction ratio (PRR) assay was conducted. Free ART, as a fast-acting antimalarial, reduced viable parasites to <0.1% within 48 h; Zein_NP@ART followed with a PRR of 4.8, both showing fast-killing profiles that decreased parasitemia. Zein_NP@ART exhibited a slightly increased parasite clearance time (PCT) but an unchanged lag phase compared to free ART, with PCT similar to the nanocarrier alone, confirming ART entrapment did not impair its antimalarial activity. This indicates Zein_NP@ART not only prolongs ART’s half-life via sustained release but also retains relatively rapid antimalarial activity, likely due to improved bioavailability.

The ring-stage survival assay (RSA) is one of the reference methods for evaluating *Plasmodium’s* partial resistance to artemisinin. Its experimental principle lies in simulating the *in vivo* pharmacokinetics and drug exposure profiles of artemisinin. Following evaluations of this protocol, we made appropriate adjustments based on the pharmacokinetic characteristics of the nanocarrier, modifying the drug concentration and exposure duration to match the target exposure profile. The results indicated that due to the sustained-release property of the nanocarrier, the efficacy of killing ring-stage parasites of resistant strains after the standard exposure duration was relatively inferior to that of free drugs. However, after the adjustments, the survival rate of 0–3 h post-invasion ring-stage *Plasmodium* was significantly reduced, which could effectively overcome ART resistance.

Meanwhile, malaria recrudescence following ART treatment is also a well-documented clinical manifestation of artemisinin resistance. Therefore, after confirming the activity of zein-based nanocarriers against resistant *Plasmodium* strains, we further employed a recrudescence assay to verify their inhibitory effect on the recrudescence of these resistant strains *in vitro*. The results demonstrated that treatment with the nanocarriers significantly suppressed the recrudescence of strains exhibiting varying degrees of ART resistance; this finding is clinically meaningful as recrudescence not only indicates treatment failure but also facilitates the transmission of resistance genes via gametocytes, and effective suppression of recrudescence thus supports the potential of zein-based nanocarriers to mitigate the spread of ART resistance and reinforce malaria control strategies.

The results of the *in vivo* experiments demonstrated that the nanocarriers exhibited markedly superior antimalarial activity when administered at 12-h intervals. This dosing regimen aligns with the pharmacokinetic characteristics of ART derivatives and the developmental cycle of *Plasmodium*, as 12-h intervals help maintain stable therapeutic drug concentrations in the bloodstream. Meanwhile, owing to their favorable aqueous solubility and enhanced oral bioavailability, the efficacy of oral administration of the nanocarrier formulation was significantly superior to that of free drugs. Free ART and its derivatives are inherently hydrophobic, leading to poor dissolution in the gastrointestinal tract and extensive first-pass metabolism, which are two key factors limiting their bioavailability. In contrast, nanocarriers act as protective reservoirs that improve drug solubility and facilitate intestinal absorption, ultimately increasing the fraction of drug reaching systemic circulation. This improvement in oral efficacy is clinically meaningful: oral delivery is preferred for malaria treatment in resource-limited settings due to its convenience and low administration burden, and nanocarrier-based formulations address the longstanding challenge of poor bioavailability for hydrophobic antimalarials.

Notably, beyond the conventional *in vivo* efficacy evaluation based on rodent malaria models, we have further extended this assessment to a humanized mouse model, and to our knowledge, this is the first attempt to investigate the effect of nanoformulations using such a model. A core advantage of this model is its ability to closely mimic human *Plasmodium* infection as human-derived erythrocytes within the model support efficient infection by human *Plasmodium* strains (48). These erythrocytes recapitulate the *in vivo* environment of human malaria more accurately than conventional animal models and thereby provide more clinically translatable drug efficacy data. However, the model still has potential limitations. For instance, two main types of humanized mouse models are currently available, and the first type spontaneously produces human erythrocytes susceptible to *P. falciparum* infection. We attempted to evaluate our nanoformulations using this model and observed successful parasite infection, but the percentage of human erythrocytes and the parasite infection rate were low (Data not shown). The lifespan of these mice was also relatively short, which made it insufficient for comprehensive antimalarial efficacy evaluation. We therefore switched to a second humanized model relying on human erythrocyte engraftment and achieved promising results, although several technical challenges remain (49). First, regular transfusion of human erythrocytes is mandatory to maintain the model, and this requirement makes the overall experimental cost higher than that of standard animal models. Second, human erythrocytes have a short survival time in mice, which means daily transfusions are needed to maintain a sufficient percentage of human erythrocytes for effective parasite infection. This demand not only increases the experimental workload but also limits long-term pharmacodynamic observations. Furthermore, the immunodeficient background of these mice results in a lack of a functional immune system that fails to replicate the human immune response to *Plasmodium* infection or the critical interplay between drugs and immunity (50, 51). Both of these factors are key to influencing clinical malaria outcomes. Finally, significant differences in drug metabolism pathways and physiological processes between mice and humans may lead to discrepancies in drug metabolism rates, toxicity profiles, and efficacy when comparing the model with clinical settings (52).

Collectively, these findings highlight that nanocarriers simultaneously optimize dosing convenience and therapeutic performance, supporting their potential to reinforce malaria control, especially in regions burdened by drug-resistant strains. However, substantial efforts can still be made to further optimize this nanoformulation and overcome its potential limitations. For instance, zein exhibits an isoelectric point at approximately pH 6.5, while at this pH, the protein carries a near-neutral net charge, leading to reduced electrostatic repulsion between particles and poor aqueous dispersibility in water. Therefore, we have to use pectin to form a complex with zein via electrostatic interactions to improve the dispersibility. Besides, studies have indicated that when such nanoformulations are applied *in vivo*, they are susceptible to sequestration by the reticuloendothelial system, which may impair the targeted delivery efficiency of encapsulated drugs and substantially reduce their *in vivo* bioavailability. Additionally, our nanoformulation exhibits limitations in mediating site-specific drug delivery and achieving controlled release profiles, as it cannot precisely trigger drug release at the designated target sites. Specifically, this nanoformulation only improves the pharmacokinetic profile and solubility of ART without exerting target responsiveness to parasite-infected erythrocytes. Thus, surface modifications should be investigated and attempted to enable the targeted delivery of ART to parasites. Moreover, the desolvation method is associated with inherent limitations, including the potential for residual solvent contamination and poor adaptability to large-scale industrial production due to cumbersome solvent removal steps and challenges in process scaling up. As a reference, albumin-based nanocarriers can be efficiently synthesized on a large scale via the Nab technology, which leverages albumin’s intrinsic amphiphilic properties for self-assembly and avoids the need for excessive organic solvents. Given the technical advantages of Nab technology in addressing the drawbacks of desolvation, further systematic exploration to refine the synthesis parameters is warranted in subsequent studies to enhance the reproducibility, stability, and pharmaceutical performance of our nanoformulation.

### Conclusion

This study reports a zein-based sustained-release nanoformulation for the antimalarial drug ART, which effectively addresses the challenges associated with traditional ART therapy, including low solubility, insufficient sustained-release capability, and compromised efficacy against drug-resistant *Plasmodium* strains. Through the design of the nanocarrier, the solubility of ART was enhanced by approximately 200-fold. This critical improvement not only promotes favorable oral absorption but also markedly boosts the drug’s *in vitro* and *in vivo* bioavailability and stability. The Zein_NP@ART nanoformulation exhibited superior physicochemical properties, including optimal particle size and zeta potential, ensuring excellent dispersibility and long-term storage stability across different pH environments. Additionally, it achieved outstanding sustained-release performance that lays the foundation for optimized pharmacokinetic behavior. *In vitro* and *in vivo* antimalarial experiments confirmed that Zein_NP@ART not only maintained the antimalarial activity of ART but also exhibited enhanced efficacy against drug-resistant *Plasmodium* strains. This improvement is attributed to the sustained-release characteristics, which significantly extended the drug’s plasma half-life. The extended half-life ensured continuous therapeutic drug release and maintained effective plasma concentrations of ART *in vivo* for prolonged durations, thereby enabling effective killing of resistant *Plasmodium* strains and suppressing their recrudescence. This approach reduced the frequency of dosing and minimized the risk of potential side effects. In humanized mouse models, the nanoformulation group exhibited significantly superior antimalarial effects compared to the free drug group, particularly against resistant strains. Generally, these results have supported our hypothesis and indicate that the zein-based sustained-release nanoformulation not only improves the solubility and sustained-release capabilities of ART but also significantly enhances its antimalarial efficacy by boosting bioavailability via improved solubility and prolonged plasma half-life. Its remarkable ability to eliminate resistant strains and inhibit their recrudescence further highlights its substantial potential for clinical translation in combating drug-resistant parasites.

## MATERIALS AND METHODS

### Parasite strains

*P. falciparum* parasite strains used in the study, including 3D7 (WT, drug-sensitive strain), 3D7^Y493H^, 803, and 3D7^C580Y^ (parasite line with low, medium, and high ART resistance), were adapted and cultured in our laboratory. Rodent malaria parasite strains, including *P. berghei* (artemisinin sensitive and resistant) and *P. chabaudi,* were cultured and stored in our laboratory.

### Methods

### Fabrication of Zein_NP@ART

The scheme of the synthesis of Zein_NP@ART is shown in Fig. 1A. Initially, ART (Aladdin) was dispersed in 100 mL of ethanol (Sinopharm Group, China), and pectin (Sigma-Aldrich) was dispersed in 100 mL of distilled water. Following ultrasonication for 30 min, respective solutions of artemisinin and pectin were obtained. Subsequently, to adjust the ethanol content to approximately 60% (vol/vol), 66.7 mL of water was integrated, followed by the addition of an accurate quantity of zein (Sigma-Aldrich) and another 30 min of ultrasonication, to prepare the zein and ART solution. This prepared solution was then amalgamated into the pectin solution and subjected to magnetic stirring for 2 h. A Zein_NP@ART dispersion was acquired post-rotary evaporation at 50 °C for 20 min. The Zein_NP@ART was finalized through freeze-drying. In the synthesis of Zein_NP@ART, variable ratios of zein to pectin and zein to artemisinin were examined to optimize the formulation.

### Characterization of Zein_NP@ART

#### Particle size and zeta potential measurement of Zein_NP@ART

Dynamic light scattering (DLS) was applied to investigate the particle size of the corresponding nanoparticles. Samples were diluted to the equivalent of 0.5 mg/mL ART. The ζ-potential and the particle size of Zein_NP@ART with different content at various pH levels were measured by a Zetasizer Nano ZS90 instrument (Malvern Instruments Ltd., Worcester Shire, UK) with scatter angle and temperature set as 173 °C and 25 °C. The ζ-potentials and the particle size of Zein_NP@ART were measured in the scope of pH 3–9, the ζ-potentials and the particle size of Zein_NP@ART with different contents at pH 2 were measured. All samples were analyzed in triplicate. The physical stability was also observed by DLS to observe the possible size enlargement. Samples were dissolved in Milli-Q water and stored at 4 °C, and taken out at regular intervals for size and ζ-potential measurement.

#### Fourier transform infrared spectrophotometric (FTIR) analysis

The FTIR a of ART, zein, pectin, and Zein_NP@ART were measured by a Fourier Transform Infrared Spectrometer (Thermo Nicolet Corporation, Madison, WI) at a resolution of 2 cm^−1^ in the range of 4,000–600 cm^−1^ with 32 scans.

#### Morphology analysis

The morphology for free ART and prepared nanoparticles was studied by SEM. In the sample preparation for SEM (Hitachi s-4800), the dispersion (0.05 mg/mL) of ART, zein colloidal particles, and Zein_NP@ART particles were first dropped on silicon wafers. The samples were dried in an oven (40°C, 24 h) and subsequently coated with gold for SEM observation.

#### *In vitro* release study

In total, 10 mg pure ART and Zein_NP@ART particles with 10 mg ART loaded were dispersed in 50 mL PBS buffer solution (pH 2 and 7.4) at 37°C ± 0.5°C with 150 rpm stirring; 0.5 mL dispersion was retrieved at 2, 4, 12, 18, 24, 36, 48, 60, and 72 h, while 0.5 mL PBS buffer solution was added to maintain sink condition. Then, the above dispersion after filtration was analyzed by high-performance liquid chromatography (HPLC). A Zorbax Eclipse C18 reversed-phase column (4.6 × 50 mm, 5 µm, Agilent, USA) was used with mobile phase acetonitrile–water (75:25, vol/vol) to obtain working reference solutions (53).

To obtain the ART content of the Zein_NP@ART particles, 80% (vol/vol) aqueous ethanol was used to dissolve the powder by sonication for 30 min. After centrifugation at 10,800 rpm for 30 min, the supernatant was collected and analyzed by HPLC.

To quantitatively determine the intracellular concentration of released ART in detail, a modified *in vitro* release assay was performed using a buffer that mimics the physiological blood environment. Briefly, 10 mg of free ART and Zein-NP@ART (containing an equivalent amount of ART) were separately added to 10 mL of the mimic buffer, which was adjusted to a 50% erythrocyte hematocrit and supplemented with 50 g/L Albumax I, and replicates were prepared for each time point. At predetermined time intervals, the samples were centrifuged to separate the supernatant and erythrocyte pellet: the ART concentration in the supernatant was directly quantified by HPLC as mentioned above. For the erythrocyte pellet, the intracellular ART concentration was determined as follows: erythrocytes were selectively lysed by passage through a Pall 0.25 μm filter, which facilitated the release of intracellular contents. The resulting cell lysate was then subjected to quantitative HPLC analysis to determine the ART concentration.

### Determination of ART loading efficiency and encapsulation concentration in Zein_NP@ART

The drug loading efficiency and encapsulation concentration of Zein_NP@ART nanoparticles from different preparation batches were quantitatively analyzed using HPLC. Briefly, each Zein_NP@ART sample was accurately weighed and fully dissolved in 80% (vol/vol) aqueous ethanol to extract free and encapsulated ART. The actual ART content in the supernatant was quantified via HPLC against a pre-validated ART standard curve.

To guarantee experimental reliability and data reproducibility, each test sample was aliquoted into three independent portions prior to use. One portion was used for the planned experimental procedures, while the remaining two portions were subjected to duplicate HPLC re-analysis under identical conditions. This duplicate verification step was designed to confirm the stability of drug loading efficiency in Zein_NP@ART during sample handling.

#### *In vitro* hemolysis test

Human red blood cells (RBCs), provided by Wuxi Blood Center, were washed five times with PBS (centrifuged at 1,500 × *g* at 4 °C for 5 min) and pelleted for the hemolysis assay. Hemolysis assays were performed according to the previously reported methods. Briefly, washed erythrocytes were diluted with PBS to 2% vol/vol, and then the hemolytic potential of free ART and Zein-NP@ART (with an equal amount of ART) was evaluated by incubating the erythrocyte suspension with serial concentrations of the test formulations. After incubation with prepared nanoparticles for 2 h at 37 °C, the samples were centrifuged at 1,500 × *g* for 10 min, and the hemolysis was measured by determining the absorbance of the supernatants at 570 nm with the absorbance at 655 nm as a reference with U-2900 UV spectrophotometer (Hitachi, Japan). PBS and 1% Triton-X were used as negative and positive controls, respectively. Two independent tests with three technical replicates were performed each time. Hemolysis, according to released hemoglobin, was calculated as follows:

Hemolysis (%) = (Abs_sample_− Abs_PBS_)/(Abs_Triton-X_− Abs_PBS_) × 100%

#### Cytotoxicity assay

The cytotoxicity of zein particles and Zein_NP@ART particles on mammalian HepG_2_ cells was evaluated using a CCK-8 assay kit (AbMole BioScience, USA). In brief, HepG_2_ cells (∼5 × 10^3^ cells per mL) cultured in Dulbecco’s modified Eagle medium supplemented with 10% fetal bovine serum and 1% penicillin/streptomycin were seeded in 96-well plates (200 μL per well) at 37°C with 5% CO_2_. After incubation with various concentrations of nanoformulations for 48 h, 10% CCK-8 solution was added into each well and incubated for an additional 2 h. The absorbance of the color development in treated and untreated cells was measured using a SpectraMax iD3 microplate reader (Molecular Devices, USA) with 450 nm as the detection wavelength. Medium was used as a negative control for background subtraction. Two independent tests with three technical replicates were performed each time.

#### Parasite culture and *in vitro* growth inhibition assay

*P. falciparum* parasite strains, including wild-type and ART-resistant strain, were cultured with O^+^ erythrocytes (provided by Wuxi Blood Center) at 2% hematocrit in RPMI 1640 medium supplemented with 0.5% (wt/vol) Albumax I, 50 mg/L hypoxanthine, 25mM NaHCO_3_, 25 mM HEPES, and 10 mg/L gentamycin. Parasite cultures were maintained at 37 °C under 5% CO_2_, 5% O_2_, and 90% N_2_ as previously described. Parasitemia was microscopically measured based on Giemsa-stained thin blood smears.

For quantitative evaluation of the antimalarial effect resulting from the proposed nanovectors, a 3-day inhibition assay was performed. Briefly, synchronized parasites (~1% parasitemia) were incubated in a 96-well plate with free ART or Zein_NP@ART (with an equivalent amount) at varied concentrations in a total volume of 200 μL. The parasites were normally cultured for 72 h, followed by SYBR Green I staining. Briefly, parasites were lysed by adding 100 μL of lysis buffer (0.12 mg/mL saponin, 0.12% Triton X-100, 30 mM Tris-HCl, and 7.5 mM EDTA) containing 5× SYBR Green I to each well. After incubation for 2 h at room temperature in the dark, the fluorescence intensity resulting from parasites was recorded at 485 nm excitation and 535 nm emission. DMSO was used as a negative control. Two experiments with three technical replicates each time were independently conducted each time. Growth inhibition rate was normalized according to the acquired fluorescence intensity for drug-treated parasites to that of the DMSO control. The IC_50_ was acquired by nonlinear fitting in GraphPad Prism.

#### Mitochondrial membrane potential analysis

To further investigate the impact of Zein_NP@ART treatment on the mitochondrial function of drug-resistant *P. falciparum* strains, a mitochondrial membrane potential (MMP) assay was performed using the MitoProbe DilC₁ (5) kit. Briefly, synchronized trophozoite-stage parasites (initial parasitemia: ~1%) were first treated with different formulations of ART in the aqueous or entrapped in Zein_NP at a concentration of 50 nM, 200 nM, and 700 nM for 6 h under standard culture conditions. ART dissolved in DMSO solution was set as the positive control to evaluate the enhanced solubility-associated antimalarial effect. After incubation, parasites were stained with DilC1 at 37°C for 30 min in the dark, and the mean fluorescence intensity was recorded by SpectraMax iD3 microplate reader using 488 nm and 640 nm as the excitation wavelength, respectively. Two independent assays with three technical replicates were performed each time.

#### Determination of ROS generation

To quantify the intracellular ROS levels in *Plasmodium* parasites following different treatments, the cell-permeable fluorescent probe 2',7'-dichlorodihydrofluorescein diacetate (DCFH-DA) was used. Briefly, synchronized trophozoite-stage parasites (initial parasitemia: ~1%) were first treated with different formulations of ART in the aqueous or entrapped in Zein_NP at a concentration of 50 nM, 200 nM, and 700 nM for 6 h under standard culture conditions. ART solved in DMSO solution was set as the positive control to evaluate the enhanced solubility-associated antimalarial effect. After treatment, parasites were incubated with 20 μM dichlorodihydrofluorescein diacetate at 37°C for 30 min in the dark. Subsequently, the parasites were harvested by centrifugation at 2,500 rpm for 5 min and thoroughly washed with PBS. Parasites were resuspended in PBS, and the fluorescence of DCF was recorded by microplate reader at an excitation wavelength of 485 nm and an emission wavelength of 520 nm. The relative ROS level was calculated by normalizing the DCF fluorescence intensity of each treatment group to that of the untreated negative control. Two independent assays with three technical replicates were performed each time.

#### Pharmacokinetic assay

Eighteen male BALB/c mice between 6 and 8 weeks of age were purchased from Vital River Laboratory Animal Technology (Beijing, China) and cultured under standardized specific pathogen-free (SPF) conditions (temperature: 22°C ± 2°C, relative humidity: 50% ± 10%, 12 h light/dark cycle) with free access to food and water. Mice were either orally or intraperitoneally administered with Zein_NP@ART at the dosage of 50 mg/kg. Blood samples were collected at 0.25, 0.5, 1, 2, 4, 6, 8, and 24 h post-injection and centrifuged at 2,500 × *g* for 4 min for plasma collection. LC-MS/MS analysis was performed according to reported conditions. Plasma concentration of artemisinin was detected for each time point, and the pharmacokinetic parameters were analyzed by WinNonlin.

#### *In vitro* parasite reduction ratio (PRR) assay

The PRR assay was performed as described previously with slight modifications regarding the measurement of parasite growth. Briefly, late-stage parasites were purified by MACS column, followed by 3-h normal culturing. The parasites were further synchronized with 5% sorbitol for 10 min at 37°C to obtain a predominant population for ring-stage parasites at 0.5% parasitemia and 1.25% hematocrit. Parasites were incubated with selected drug formulations at the concentration of 10× IC_50_ (100 nM for atovaquone) in 6-well plates, and the drug-containing medium was replenished every 24 h. Cultures at corresponding time points (0, 24, 48, 72, 96, and 120 h) were aliquoted and serially diluted into 96-well plates after washing three times with medium. Each sample was seeded with four replicates and then incubated for up to 28 days under standard conditions for parasitemia monitoring. On days 21 and 28, parasite viability measurement was performed by HRP-2-based ELISA, and key parameters, including Lag phase, log (PRR), and PCT, were calculated for *in vitro* mode-of-action determination.

#### Ring-stage survival assays

*In vitro* survival assay was performed to investigate the resistance phenotype (47). Briefly, resistant parasite strains were twice synchronized by 5% sorbitol at 40 h interval, followed by 40%/70% Percoll treatment. Then parasites were accurately cultured for 3 h with fresh erythrocytes to allow merozoite invasion. Afterward, remaining late-stage parasites were eliminated by 5% sorbitol to obtain 0–3 h ring stage parasites. Finally, cultures were dispensed into 24-well plates at 0.5% parasitemia and 2% hematocrit.

According to the pharmacokinetic assay, we slightly modified the standard RSA protocol by altering the dosage and period of incubation. Parasites exposed to 700 nM artemisinin for 6 h were set as the control. Parasites were divided into four groups that received the following treatments: (i) Zein_NP@ART1: 6 h 450 nM ART (entrapped in Zein nanoparticles) incubation, (ii) Zein_NP@ART2: 6 h 650 nM ART (entrapped in Zein nanoparticles) incubation, (iii) Zein_NP@ART3: 20 h 450 nM ART (entrapped in Zein nanoparticles) incubation, and (iv) Zein_NP@ART4: 22 h 650 nM ART (entrapped in Zein nanoparticles) incubation. After incubation for the desired period, cultures were washed twice with RPMI 1640 incomplete media to remove excess drugs and returned to standard culture conditions for 66 h in new wells. Survival rates were microscopically assessed by counting the percentage of viable parasites that entered the second-round life cycle with normal trophozoite or ring morphology (~10,000 erythrocytes).

#### Recrudescence assay

The recrudescence protocol was performed according to the previously reported method with slight modifications. Briefly, unsynchronized parasites in 15 mL cultures (2% parasitemia and 5% hematocrit) were exposed to different exposures of corresponding formulations of artemisinin at selected intervals. Parallel cultures exposed to the DMSO vehicle were set as blank control. The exposure concentration and period for nanocarriers were set as 450 nM for 20 h and 650 nM for 22 h, based on the results from the pharmacokinetic assay. Parasites exposed to 700 nM artemisinin for 6 h were set as the control. After each exposure, the parasite cultures were washed twice with the medium to remove the drug or vehicle concentration. To prevent potential hemolysis, 200 µL of freshly prepared RBCs at 50% hematocrit was added to the culture on a weekly basis. On the selected follow-up days (1, 2, 3, 4, 7, 9, 11, 14, 16, 18, 21, 23, 25, 28, and 30), parasitemia was microscopically checked by Giemsa’s solution-stained thin smears. Cultures were maintained under standard culturing conditions until parasitemia reached ~2%.

#### *In vivo* antimalarial assay in rodent model

*In vivo* antimalarial activity was primarily evaluated in either *P. berghei-* or *P. chabaudi-*infected rodent model according to Peters 4-day suppressive assay. Female BALB/c mice between 6 and 8 weeks of age were purchased from Vital River Laboratory Animal Technology (Beijing, China). Mice were housed under strict pathogen-free conditions at an ambient temperature (22°C ± 2°C) and 40%–70% humidity. All animals were provided with food and autoclaved tap water *ad libitum* and acclimated for 5 days before the experiment initiation. Mice were intraperitoneally infected with 200 μL of parasite-infected red blood cells (iRBCs) obtained from donor mice, with a density of 2 × 10⁷ parasitized cells per mL. After infection, parasitemia was monitored daily via Giemsa-stained thin blood smears, and drug treatment was initiated when the parasitemia reached 2%–5%. Prior to treatment, mice were randomly divided into experimental groups (*n* = 5 per group) to evaluate the efficacy of different ART formulations and administration routes. Due to the differential absorption profiles between intraperitoneal (i.p.) injection and oral administration, the oral doses were appropriately adjusted based on pharmacokinetic data. Mice were divided into the following groups that received different treatment.

Intraperitoneal injection group: Mice (*n* = 5 per group) were administered with different ART formulations at doses of 2.5 mg/kg/day or 5.0 mg/kg/day (based on equivalent ART amount), for 4 consecutive days with an administration interval of 12 h or 24 h.

Oral administration group: Mice (*n* = 5 per group) were administered with the different ART formulations via oral gavage at doses of 5.0 mg/kg/day or 10.0 mg/kg/day (based on equivalent ART amount), for 4 consecutive days with an administration interval of 12 h or 24 h

The negative control group received an equal volume of 0.9% saline injection. Parasitemia of infected mice was monitored by microscopic examination of methanol-fixed thin blood smears stained with Giemsa.

#### *In vivo* antimalarial assay in humanized mice model

To further investigate the *in vivo* antimalarial activity against *P. falciparum*-sensitive and -resistant strains, a humanized mouse model with the capability of *P. falciparum* infection was further employed. Female NCG-X mice (NOD/ShiLtJGpt-*Prkdc*^em26Cd52^*Il2rg*^em26Cd22^*kit^em1Cin(V831M)^*/Gpt mice) aged ~10 weeks were purchased from GemPharmatech (Nanjing, China) for human erythrocytes engraftment. Mice were cultured as described above. Human O^+^ erythrocytes without malaria infection history were obtained from Wuxi Blood Center. The erythrocytes were washed twice with RPMI-1640 medium at room temperature and stored at 4°C until use. Prior to intraperitoneal injection, the blood suspension was warmed at 37°C for 20 min to ensure physiological compatibility with the recipient animals. Mice were intraperitoneally injected with 1 mL prewarmed hRBC suspension (50% hematocrit in RPMI medium) every day according to standard engraftment protocol. The engraftment efficiency was monitored by measuring the erythrocyte percentage in which hRBC was stained by PE-Cyanine7 labeled anti-human CD235a mAb (Invitrogen), and mRBC was stained by anti-mouse TER-119 mAb (BioLegend). Briefly, anti-human CD235a mAb was diluted to a final ratio of 1:1,000, and anti-mouse TER-119 mAb was diluted to 1:750. Blood samples were collected from the tail vein and mixed with 50 μL of DPBS solution. A 1 μL aliquot of the blood-DPBS mixture was aliquoted and stained with 100 μL of pre-prepared staining buffer for 15 min on ice in the dark. After staining, samples were supplemented with 200 μL of DPBS and then subjected to flow cytometry analysis to determine the engraftment efficiency by quantifying the percentage of human red blood cells (hRBCs). Mice with peripheral stable engraftment efficiency (hRBC levels were ~70%) were infected with *in vitro* cultured *P. falciparum* strains (WT or C580Y mutant strain) at a density of 1 × 10^7^ parasites per mouse. After 4 days of consecutive infection, mice were randomized into different groups (*n* = 3 per group) for drug treatment. Our pre-experimental data (not shown) demonstrated that, partly due to the physical condition of immune-deficient mice, the percentage of hRBCs maintains stability in the early phase post-engraftment but begins to undergo gradual reduction after 4 weeks. Such a decrease in hRBC proportion would significantly compromise the infection efficiency and persistent propagation of *P. falciparum*, which in turn would distort the accurate assessment of antimalarial efficacy. To address this confounding factor, the total duration of the current experiment was specifically designed as 23 days, a period that ensures the observation extends to 1 week after the drug treatment, thereby enabling comprehensive and reliable evaluation of the antimalarial effect. The antimalarial activity and the recrudescence assay were further assessed the day after the last drug treatment (D8) and at the end of the observation period (D14). Blood was collected from the tail vein, and parasitemia was assessed daily by microscopic examination. The therapeutic efficacy of the treatment was evaluated comprehensively based on two key parameters: the parasite inhibition rate at the predefined time point, and the dynamic daily parasitemia profile monitored throughout the experimental period. This dual-parameter evaluation strategy ensures a rigorous and holistic characterization of the treatment’s antimalarial performance, capturing both immediate efficacy and long-term control of parasite proliferation.

### Statistical analysis

Statistical analysis was performed by Student’s *t*-test and one-way analysis of variance (ANOVA) via GraphPad Prism 8.0 software. Differences between treated and control groups were assessed by a two-tailed Student’s *t*-test, while comparisons among multiple groups were analyzed via one-way ANOVA, followed by multiple comparison tests with Bonferroni correction. Detailed statistical information for each result was described in the corresponding figure legends. Mouse survival was monitored for 1 month, with survival curves generated and analyzed using the Kaplan-Meier method. Statistical significance was defined as *P < 0.05, **P < 0.01, ***P < 0.001.

## Data Availability

All data necessary to assess the conclusions are present in the article and the supplemental material.

## SUPPLEMENTAL MATERIAL

Data Set S1 mbio.03696-25-s0001.xlsx Source data for the figures.

Supplemental Material mbio.03696-25-s0002.pdf Supplemental methods and Fig. S1 to S5.

ASM does not own the copyrights to Supplemental Material that may be linked to, or accessed through, an article. The authors have granted ASM a non-exclusive, world-wide license to publish the Supplemental Material files. Please contact the corresponding author directly for reuse.
