# Supplementary material for: Development and evaluation of novel zein-based artemisinin sustained-release formulation for treating drug-resistant malaria
Source: mBio. 2026 Jan 12;17(2):e03696-25. doi: 10.1128/mbio.03696-25 (PMC12892940; doi:10.1128/mbio.03696-25)
Supplement: Supplemental Material — Supplemental methods and Fig. S1 to S5. [file mbio.03696-25-s0002.pdf]

Development and Evaluation of Novel Zein-Based Artemisinin Sustained-Release Formulation for Treating Drug-Resistant Malaria

Short title: Antimalarial Evaluation for Zein-Based Artemisinin Sustained-Release Formulation

Yijie Wang[1, #], Xinyu Yu[2,3, #], Xinyu Zhang[2,3, #], Xiaohui He[3], Yongxin Tang[2,3], Ling Fang[3,4], Richard Culleton[5], Qingfeng Zhang[6]*, Weifu Dong[1, *], Jun Cao[2,3,7, *]

Supporting information

Fabrication of PLGA@ART

PLGA (2 g) and ART (1 g) were dissolved in 50 mL of acetone and sonicated gently until a clear solution was obtained as an organic phase. A: 1% PVA solution (200 mL) was prepared and stirred at 800 rpm at room temperature as an aqueous phase.

The organic phase was injected into the aqueous phase at a rate of 0.5 mL/min via a syringe pump, with constant stirring at 400 rpm and a reaction temperature maintained at 40 ° C throughout the addition process. Upon complete addition, stirring was continued for 30 minutes. Acetone was subsequently removed by rotary evaporation at 65 ° C for 30-60 minutes. The total volume of the system was adjusted back to approximately 15 mL using ultrapure water, and the resulting nanoparticle suspension was centrifuged at 15,000×g for 10 minutes. The supernatant was discarded, and the pellet was resuspended in 10 mL of ultrapure water, this washing step was repeated 2–3 times. Finally, the nanoparticles were collected and freeze-dried.

Fabrication of LIPO@ART

Phosphatidylcholine (4 g), cholesterol (1 g), artemisinin (ART, 1 g), and PEG (0.4 g, MW=2000) were sequentially dissolved in 50 mL of anhydrous ethanol, and the mixture was stirred at 400rpm and for 20 min until a clear solution was obtained.

The obtained solution was then added into 50 mL of water using a syringe pump at a rate of 0.5 mL/min, maintaining gentle stirring at 400 rpm, 40 °C during addition. After the addition was complete, stirring was continued for an additional 30 min to allow initial self-assembly of liposomes. Ethanol was subsequently removed by rotary evaporation(45°C, 15 min), and the suspension was centrifuged at 4000 rpm for 5 min. The supernatant was collected, and the volume was concentrated to approximately 20-40 mL.

Determination of ART loading efficiency and encapsulation concentration in PLGA_NP@ART and LIPO_NP@ART

The drug loading efficiency and encapsulation concentration of Zein_NP@ART nanoparticles from different preparation batches were quantitatively analyzed using high-performance liquid chromatography (HPLC). Briefly, each Zein_NP@ART

sample was accurately weighed and fully dissolved in 80% (v/v) aqueous ethanol to extract free and encapsulated ART. The actual ART content in the supernatant was quantified via HPLC against a pre-validated ART standard curve.

To guarantee experimental reliability and data reproducibility, each test sample was aliquoted into three independent portions prior to use. One portion was used for the planned experimental procedures, while the remaining two portions were subjected to duplicate HPLC re-analysis under identical conditions. This duplicate verification step was designed to confirm the stability of drug loading efficiency in nanoformulations during sample handling.

Drug pressure selection and development of resistance. *P. berghei* ART-resistant strains were generated via a repeated drug selection protocol as previously described (1, 2). Briefly, female BALB/c mice (6-8 weeks) were intraperitoneally infected with the parental *P. berghei* strain. Subsequently, infected mice were randomly assigned to four experimental groups (n=3): three ART treatment groups with graded dosages (1.25, 2.5, and 5 mg/kg body weight) and one vehicle control group. All treatments were repeated for 4 consecutive days, consistent with the standard 4-day suppressive test for antimalarial drug evaluation. Parasitemia was quantitatively assessed by microscopic examination of Giemsa-stained thin blood smears daily. The 50% effective level (ED_{50}) and 90% effective level (ED_{90}) against each parasite line were calculated using the linear regression model based on the parasite inhibition rate. The resistance index (I_{90}) was defined as the ratio of ED_{90} values between the drug-selected resistant strain and the parental sensitive strain. According to previously established criteria (3), parasite strains were classified into four resistance categories based on I_{90} values: sensitive (I_{90} = 1.0), slight resistance (I_{90} = 1.01-10.0), moderate resistance (I_{90} = 10.01-100.0), and high resistance ($I_{90} > 100.0$).

After determining the ED_{50} and ED_{90} of the parental parasite strain, an additional 3 female BALB/c mice were intraperitoneally infected with the same parental strain. Parasitemia was quantitatively monitored via daily microscopic examination of Giemsa-stained thin blood smears until it reached 3%-5%. Then mice were treated with ART at the dosage of ED_{99} and the level of resistance was assessed after every 10

passages (10 cycles) using the 4-day suppressive test for the determination of new ED_{50} and ED_{90}.

This drug selection and resistance monitoring process was continuously repeated for 30 consecutive passages to ensure the establishment of a genetically stable ART-resistant *P. berghei* strain and the phenotype data were listed in the SourceData file (the last sheet). Subsequent to the successful generation of this resistant strain, the *in vivo* antimalarial activity of the test formulations against it was evaluated as described.

Passage No.	ED_{50} (mg/kg)	I_{50}	ED_{90} (mg/kg)	I_{90}
Parent	0.97	N.A.	3.48	N.A.
10	1.59	1.639	4.33	1.244
20	2.77	2.856	10.39	2.986
30	3.32	3.423	12.21	3.509

Reference

1. Kiboi DM, Irungu BN, Langat B, Wittlin S, Brun R, Chollet J, Abiodun O, Nganga JK, Nyambati VC, Rukunga GM, Bell A, Nzila A. 2009. Plasmodium berghei ANKA: selection of resistance to piperaquine and lumefantrine in a mouse model. Exp Parasitol 122:196-202.
2. Xiao SH, Yao JM, Utzinger J, Cai Y, Chollet J, Tanner M. 2004. Selection and reversal of Plasmodium berghei resistance in the mouse model following repeated high doses of artemether. Parasitol Res 92:215-219.
3. Merkli B, Richle R, Peters W. 1980. The inhibitory effect of a drug combination on the development of mefloquine resistance in Plasmodium berghei. Ann Trop Med Parasitol 74:1-9.

Supplementary Figures

Figure S1

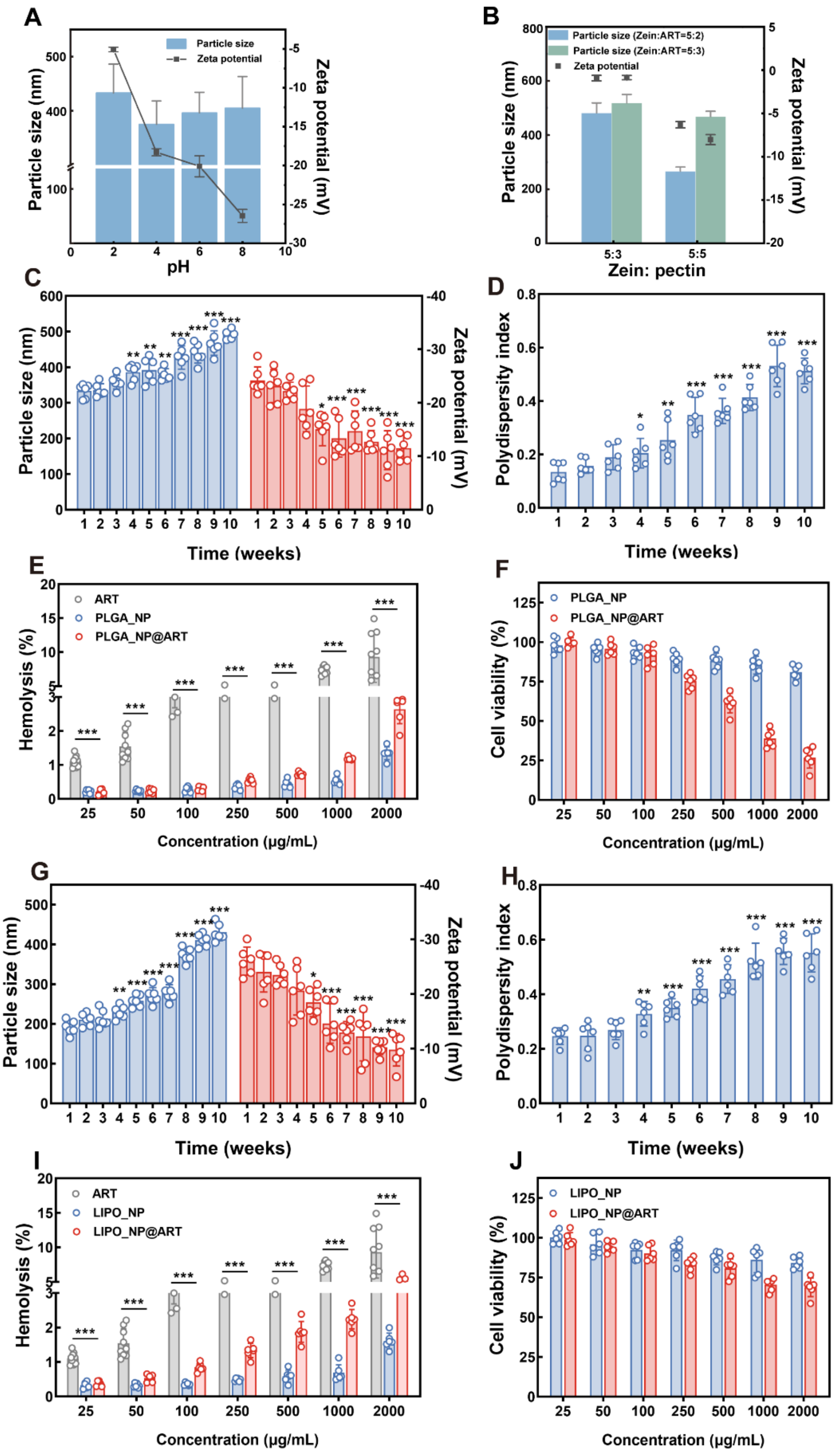

Figure. S1 Stability characterization of Zein_NP@ART, PLGA_NP@ART and LIPO_NP@ART.

(A) The particle size and zeta potential of Zein_NP@ART at different pH. **(B)** The particle size and zeta potential of Zein_NP@ART with different amounts of pectin and artemisinin added. **(C-D)** Long-term stability of nanoparticles stored at 4 °C for up to 10 weeks: **(C)** Dynamic changes in particle size (left y-axis, nm) and zeta potential (right y-axis, mV) of PLGA _NP@ART during storage; **(D)** Polydispersity index (PDI) of PLGA _NP@ART over the storage period, reflecting the temporal stability of their particle size distribution uniformity. **(E)** Hemolysis percentage of erythrocytes treated with PLGA _NP, PLGA _NP@ART, or free ART (as control) at concentrations ranging from 25 to 2000 μg/mL following incubation at 37°C for 2 hours, evaluating the blood compatibility of the nanocarriers. **(F)** Cytotoxicity of PLGA _NP, PLGA _NP@ART on HepG2 cell viability, determined by CCK-8 assay at concentrations of 25-2000 μg/mL. **(G-H)** Long-term stability of nanoparticles stored at 4 °C for up to 10 weeks: **(G)** Dynamic changes in particle size (left y-axis, nm) and zeta potential (right y-axis, mV) of LIPO_NP@ART during storage; **(H)** Polydispersity index (PDI) of LIPO_NP@ART over the storage period, reflecting the temporal stability of their particle size distribution uniformity. **(I)** Hemolysis percentage of erythrocytes treated with LIPO_NP, LIPO_NP@ART, or free ART (as control) at concentrations ranging from 25 to 2000 μg/mL following incubation at 37°C for 2 hours, evaluating the blood compatibility of the nanocarriers. **(J)** Cytotoxicity of LIPO_NP, LIPO_NP@ART on HepG2 cell viability, determined by CCK-8 assay at concentrations of 25-2000 μg/mL.Data were presented as mean ± SD from two independent experiments with technical triplicates. Statistical significance was analyzed using Student's *t*-test, with all experimental groups compared with the control group. * $P < 0.05$, ** $P < 0.01$, ***$P < 0.001$.

Figure S2

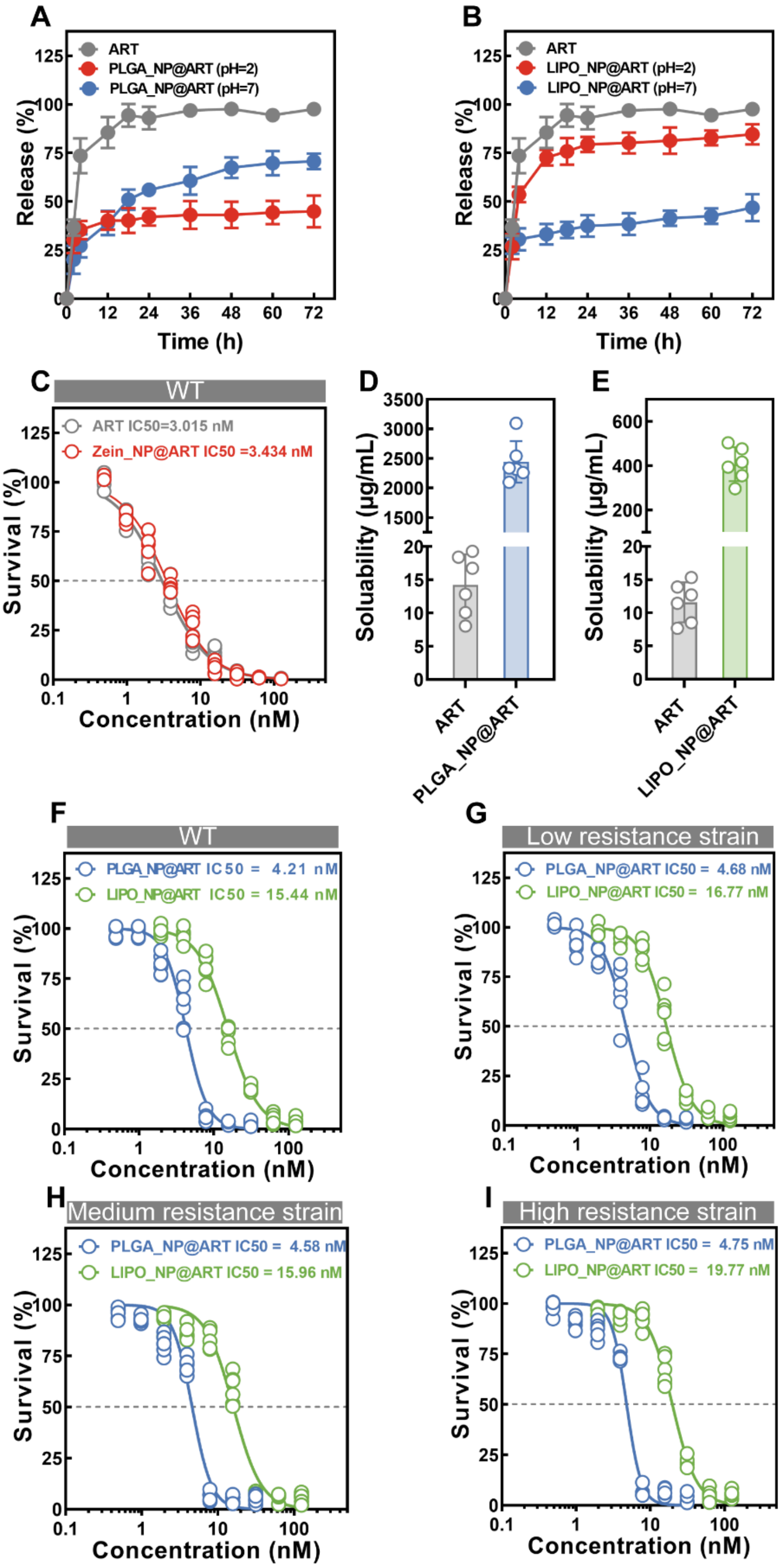

Figure. S2 *In vitro* drug release kinetics and antimalarial efficacy evaluation of PLGA_NP@ART and LIPO_NP@ART.

Cumulative release profiles of ART from PLGA_NP@ART **(A)** and LIPO_NP@ART

(B) under varying pH conditions, free ART was set as a control. **(C)** Typical dose-response evaluations of free ART and Zein_NP@ART on asynchronous cultures of *P. falciparum* 3d7 strain (WT) during the asexual stage. A 3-day SYBR Green I-based dose-response assay was performed to quantify parasite growth. The parasites were exposed to a series of concentrations for 72 hours (with an [inhibitor] vs. normalized response fit). These analyses were performed using nonlinear regression in GraphPad Prism 8.0 software. **(D-E)** The solubility of free ART and ART encapsulated in PLGA_NP@ART**(D)** and LIPO_NP@ART **(E)** in water. **(F-I)** Typical dose-response evaluations of PLGA_NP@ART and LIPO_NP@ART on asynchronous cultures of *P. falciparum* strains during the asexual stage. Data were presented as mean ± SD from two independent experiments with technical triplicates.

Figure S3

A

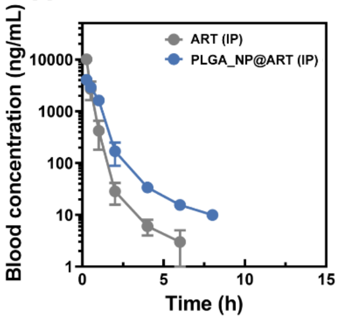

Units	Parameter	PLGA_NP@ART (IP)			Mean	SD	CV%
		Rep1	Rep2	Rep3			
hr*ng/mL	AUC0-last	3818.45	4406.16	4933.24	4385.95	557.674	12.715
hr*ng/mL	AUC0-INF	3852.52	4417.69	4956.16	4408.789	551.876	12.518
h	MRT(0-t)	0.693	0.716	0.682	0.697	0.017	2.489
h	MRT(0-∞)	0.789	0.74	0.73	0.753	0.032	4.193
hr	t1/2z	2.392	1.343	2.175	1.97	0.554	28.108
L/kg	Vz	44.798	21.935	31.662	32.798	11.474	34.983
L/h/kg	CLz	12.979	11.318	10.088	11.462	1.451	12.658
ng/mL	C0	5019.96	5263.49	6685.55	5656.331	899.607	15.904

B

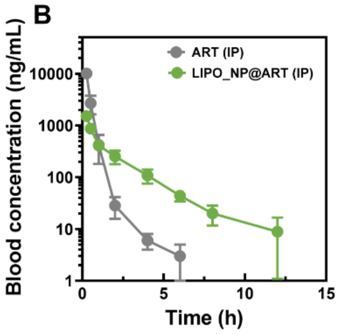

Units	Parameter	LIPO_NP@ART (IP)			Mean	SD	CV%
		Rep1	Rep2	Rep3			
hr*ng/mL	AUC0-last	2320.94	1926.38	2063.86	2103.732	200.279	9.52
hr*ng/mL	AUC0-INF	2343.7	1995.06	2083.8	2140.853	181.186	8.463
h	MRT(0-t)	1.61	1.646	1.519	1.592	0.065	4.112
h	MRT(0-∞)	1.741	2.166	1.598	1.835	0.295	16.1
hr	t1/2z	2.132	3.294	5.249	3.558	1.575	44.269
L/kg	Vz	65.646	119.137	43.249	76.011	38.991	51.297
L/h/kg	CLz	21.334	25.062	23.995	23.464	1.92	8.183
ng/mL	C0	3771.75	2190.05	2169.5	2710.436	919.185	33.913

Figure. S3 *In vitro* pharmacokinetic profiles of PLGA_NP@ART and LIPO_NP@ART.

(A) Pharmacokinetic profiles of ART and PLGA_NP@ART following intravenous administration at a dosage of 50 mg/kg; the inset table presents pharmacokinetic parameters fitted from blood drug concentration-time data. **(B)** Pharmacokinetic profiles of ART and LIPO_NP@ART following intravenous administration at a dosage of 50 mg/kg; the inset table presents pharmacokinetic parameters fitted from blood drug concentration-time data.

Figure S4

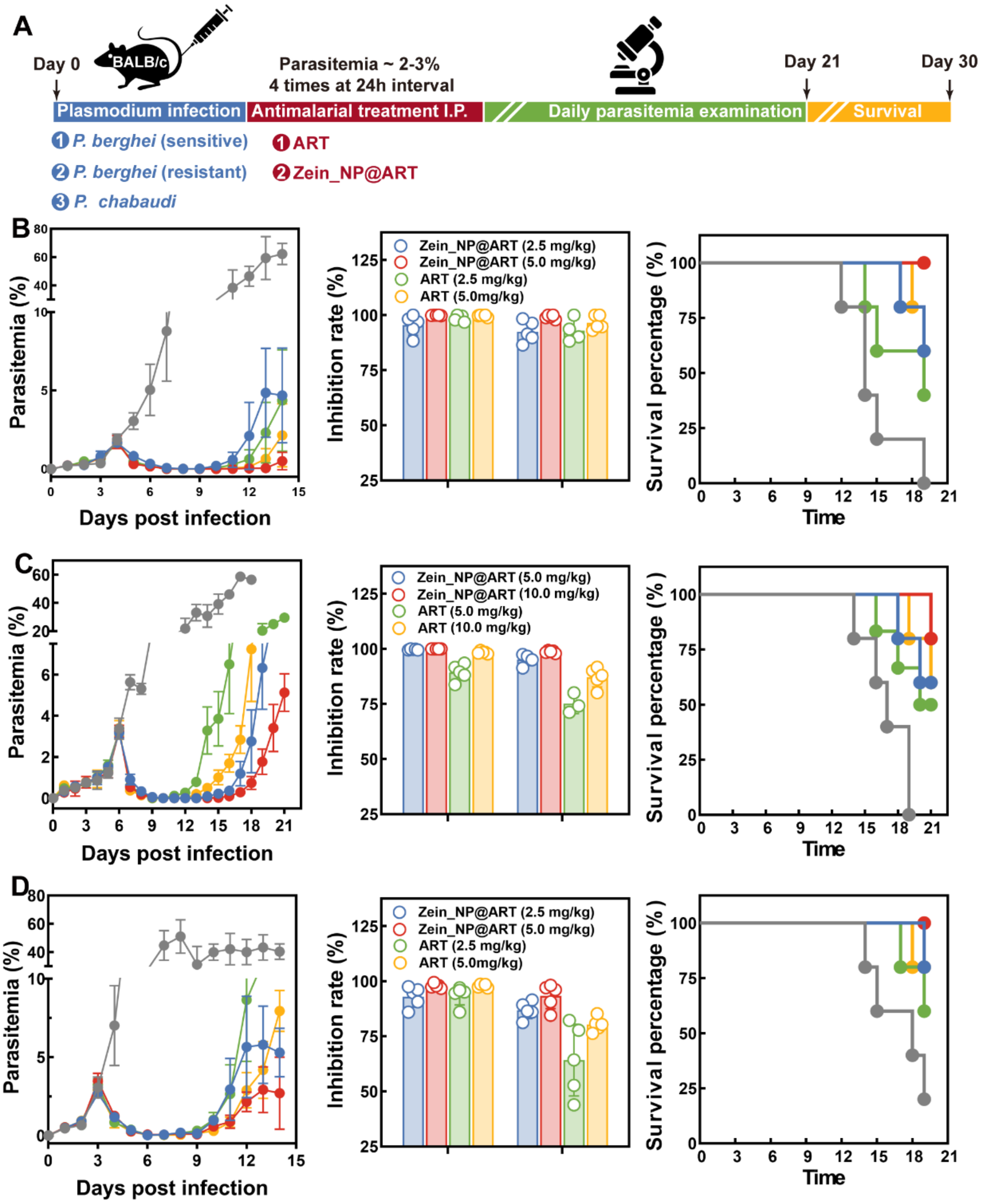

Figure. S4 *In vivo* antimalarial activity evaluation of intraperitoneal Zein_NP@ART treatment in rodent model with a 24 h interval.

(A) Schematic illustration of parasite infection and Zein_NP@ART treatment: Female mice were intraperitoneally infected with 10^6 parasites on Day 0. Antimalarial intervention was initiated on Day 4 at 24 h interval, by intraperitoneal injection of corresponding ART formulations when peripheral blood parasitemia had reached 2-5%.

Parasitemia of *P.berghei* WT strain (**B**. left panel), *P.berghei* resistant strain (**C**. left panel)and *P.chabaudi* (**D**. left panel)were determined in five experimental groups: vehicle control group, low dose free ART group, medium dose free ART group and Zein_NP@ART treated group with equal amount of ART. Parasitemia levels were quantified daily by an experienced technician via microscopic examination of Giemsa-stained blood smears from at least 5000 RBCs. Data were presented as mean ± SD with 5 mice in each group. Parasite inhibition rate for *P.berghei* WT strain (**B**. middle panel), *P.berghei* resistant strain (**C**. middle panel) and *P.chabaudi* (**D**. middle panel) infected mice administrated with different formulations of ART at corresponding time point (Due to inherent differences in parasite developmental stages and experimental endpoint definitions across groups, detailed timepoints for inhibition rate assessment are provided in the SourceData file). Kaplan-Meier survival curve for all groups of mice post infection of *P.berghei* WT strain (**B**. right panel), *P.berghei* resistant strain (**C**. right panel)and *P.chabaudi* (**D**. right panel). Survival outcomes of mice in all experimental groups were monitored continuously post-infection.

Figure S5

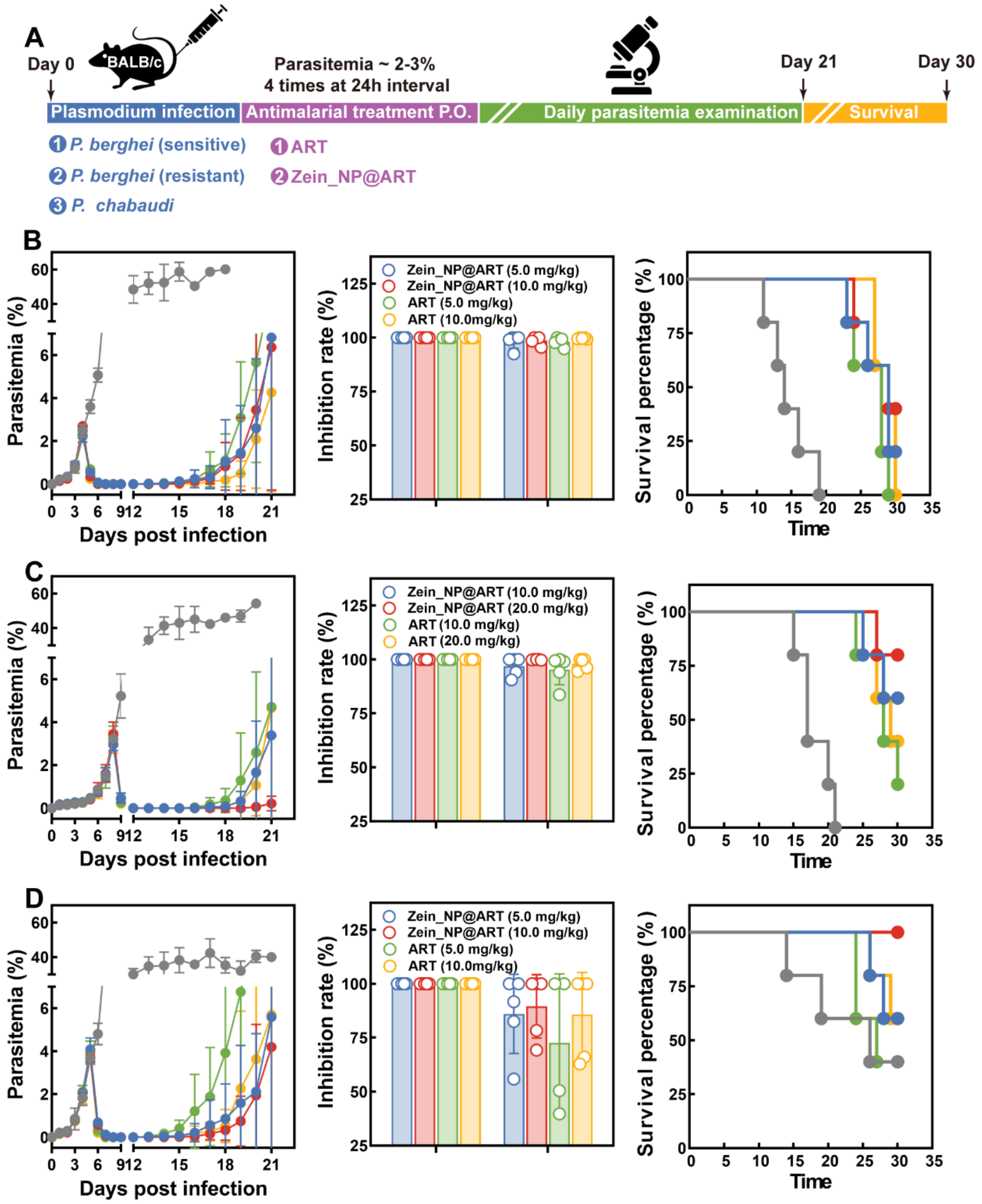

Fig. S5 *In vivo* antimalarial activity evaluation of oral Zein_NP@ART treatment in rodent model with a 24 h interval.

(A) Schematic illustration of parasite infection and Zein_NP@ART treatment: Female mice were intraperitoneally infected with 10^6 parasites on Day 0. Antimalarial intervention was initiated on Day 4 at 24 h interval, by oral administration of corresponding ART formulations when peripheral blood parasitemia had reached 2-5%.

Parasitemia of *P.berghei* WT strain (**B**. left panel), *P.berghei* resistant strain (**C**. left panel)and *P.chabaudi* (**D**. left panel)were determined in five experimental groups: vehicle control group, low dose free ART group, medium dose free ART group and Zein_NP@ART treated group with equal amount of ART. Parasitemia levels were quantified daily by an experienced technician via microscopic examination of Giemsa-stained blood smears from at least 5000 RBCs. Data were presented as mean ± SD with 5 mice in each group. Parasite inhibition rate for *P.berghei* WT strain (B. middle panel), *P.berghei* resistant strain (**C**. middle panel) and *P.chabaudi* (**D**. middle panel) infected mice administrated with different formulations of ART at corresponding time point (Due to inherent differences in parasite developmental stages and experimental endpoint definitions across groups, detailed timepoints for inhibition rate assessment are provided in the SourceData file). Kaplan-Meier survival curve for all groups of mice post infection of *P.berghei* WT strain (**B**. right panel), *P.berghei* resistant strain (**C**. right panel)and *P.chabaudi* (**D**. right panel). Survival outcomes of mice in all experimental groups were monitored continuously post-infection.
